# Immunomodulatory Mechanisms of Chronic Wound Healing: Translational and Clinical Relevance

**DOI:** 10.1002/mco2.70378

**Published:** 2025-10-20

**Authors:** Mahrukh Riaz, Muhammad Zohaib Iqbal, Agnes S. Klar, Thomas Biedermann

**Affiliations:** ^1^ Tissue Biology Research Unit Department of Surgery University Children's Hospital Zurich Zurich Switzerland; ^2^ Children's Research Center University Children's Hospital Zurich Zurich Switzerland; ^3^ University of Zurich Zurich Switzerland

**Keywords:** biomaterials, chronic wound, clinical trials, EVs, immunomodulation, stem cells

## Abstract

Chronic wounds—such as diabetic foot ulcers (DFUs), pressure ulcers (PUs), and venous leg ulcers (VLUs)—pose a serious clinical challenge due to their prolonged inflammatory phase and impaired healing. Increasing evidence reveals that dysregulated immune responses are central to the pathogenesis of chronic wounds. A complex interplay between innate and adaptive immune cells, including macrophages, neutrophils, and T cells, contributes to chronic inflammation, extracellular matrix (ECM) degradation, and tissue repair failure. While current treatments target symptoms, they often overlook the underlying immunopathology. This review provides a comprehensive analysis of the immunomodulatory mechanisms governing chronic wound healing, emphasizing the distinct immune landscapes in DFUs, PUs and VLUs. It explores immunotherapeutic strategies including cytokine‐based therapies, protease inhibitors, and biomaterials with immunoregulatory functions. Special attention is given to the emerging roles of mesenchymal stem cells (MSCs) and MSC‐derived extracellular vesicles (EVs) in modulating inflammation, promoting angiogenesis, and enhancing tissue regeneration. Recent clinical trials of these therapies are also critically evaluated to bridge preclinical findings with translational relevance. By integrating immunology, regenerative medicine, and clinical insights, this review highlights novel targets and strategies for immunomodulation, providing a valuable framework for advancing precision therapies in chronic wound care.

## Graphical abstract

This graphical abstract illustrates the immunomodulation of chronic wounds through various therapeutic approaches aimed at regulating the immune response to promote healing.

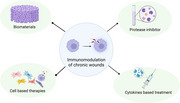

## 1 Introduction

Wounds that fail to heal within a month are classified as chronic wounds or ulcers, characterized by prolonged inflammation [1]. Chronic wounds are a severe condition affecting approximately 4% of the global population, with over 10 million cases in Europe alone, costing healthcare systems over €4 billion annually. They are most prevalent in individuals over 65 and are often linked to chronic diseases like diabetes, vascular disorders, heart disease, and obesity. If left untreated, they can lead to severe infections and limb necrosis, necessitating surgical intervention [2]. In modern healthcare, the staggering reality that 70% of amputations result from unhealed wounds highlights the urgent need for effective interventions. In the United States alone, approximately 6 million individuals suffer from nonhealing wounds, contributing to an overwhelming healthcare cost of nearly $25 billion. Moreover, chronic wounds significantly reduce quality of life, creating health, social, and economic burdens for patients and their families. With rising risk factors and an aging population, their prevalence is expected to increase. Different ulcer types arise from underlying conditions: VLUs result from chronic venous insufficiency, PUs develop in bedridden patients, and diabetic foot ulcers (DFUs) are prevalent in those with diabetes and obesity [1]. Given these alarming statistics, there is a pressing demand for innovative research and therapeutic strategies to alleviate both the human and economic burden of chronic wounds [3].

Wound healing is a complex process that is critically regulated by immune responses, particularly through the actions of cytokines, chemokines, and the interplay of various immune cells. Wound healing proceeds through several overlapping phases: hemostasis, inflammation, proliferation, and remodeling. Each phase is essential and heavily influenced by the immune system, which orchestrates a complex response to injury. The inflammatory phase is an essential response to tissue injury, which serves to eliminate pathogens and facilitate the subsequent healing processes. Inflammation is characterized by the recruitment of neutrophils and macrophages to the wound site. Neutrophils are generally the first responders, delivering proteolytic enzymes and reactive oxygen species (ROS) to combat potential infections [4]. Following this initial influx, macrophages assume a central role by transitioning from a proinflammatory (M1) to an anti‐inflammatory (M2) phenotype, which is crucial for resolving inflammation and promoting tissue repair [5, 6]. M1 macrophages are associated with the release of proinflammatory cytokines like tumor necrosis factor‐α (TNF‐α) and IL‐1β, whereas M2 macrophages promote anti‐inflammatory signals and tissue repair processes [6, 7]. Excessive or chronic inflammation can hinder wound healing by disrupting neovascularization and the function of essential repair cells [5, 8]. Therefore, a tightly regulated inflammatory response is necessary. For instance, studies have shown that the balance between proinflammatory and anti‐inflammatory cytokines determines the transition to the proliferative phase. Inadequate resolution of inflammation can lead to chronic wounds, characterized by persistent immune cell activity and delayed healing [8]. This evolving understanding has spurred significant interest in exploring immunomodulatory therapies as promising strategies to shift the wound microenvironment from a destructive to a reparative state. In addition to macrophages and neutrophils, other immune cells and fascia fibroblasts play significant roles in wound healing. T‐cells, for instance, contribute to immune surveillance and cytokine production, essential for proper wound repair [9, 10]. Fascia fibroblasts, a specialized subset of fibroblasts located within the fascia, play a critical role in wound healing by contributing to the formation of the ECM and modulating the inflammatory response. They secrete various cytokines and growth factors, including transforming growth factor‐beta (TGF‐β), that promote fibroblast activity and modulate the activity of macrophages and other immune cells within the wound microenvironment [11, 12]. In response to injury, fascia fibroblasts can differentiate into myofibroblasts, which possess enhanced contractile properties. Myofibroblasts are responsible for wound contraction, effectively pulling the edges of the wound together as new tissue forms [13]. If fascia fibroblasts fail to transition properly through the healing phases, this may result in prolonged inflammation and impaired tissue repair [14].

Despite significant advances in wound care, chronic wounds remain a major global health burden, affecting millions of patients and contributing to high patient morbidity and, thus, healthcare costs. Traditional treatments often fail to address the underlying immune dysregulation that perpetuates chronic inflammation and impedes healing in conditions such as DFUs, PUs, and venous leg ulcers (VLUs). Recent discoveries underscore the central role of the immune system in regulating the wound healing process, from the inflammatory phase to tissue remodeling. However, this knowledge is scattered across diverse domains, including immunology, biomaterials, regenerative medicine, and clinical research. Moreover, novel immunomodulatory strategies—such as cytokine therapies, protease inhibitors, bioactive scaffolds, MSCs, and MSC‐derived EVs—are emerging as promising therapeutic options, but a comprehensive synthesis of their mechanisms and clinical translation is lacking. This review aims to bridge that gap by integrating current knowledge on immune dysregulation in different types of chronic wounds; exploring the immunomodulatory potential of innovative therapeutic approaches; and highlighting translational insights and ongoing clinical trials to support evidence‐based application. By consolidating recent advances and identifying challenges in the field, this review serves as a critical resource for researchers, clinicians, and biomedical innovators, guiding the development of targeted, immune‐focused therapies for improved chronic wound management.

Immunomodulation has emerged as a promising approach to enhance chronic wound healing by addressing the underlying immune dysfunction that often contributes to prolonged inflammation and the failure of effective healing responses. Recent advances in immunobiology and regenerative medicine have shed light on novel therapeutic strategies aimed at restoring immune homeostasis in chronic wounds. This review provides a detailed examination of the immunological landscape of chronic wound healing, beginning with an overview of the distinct wound healing stages and the dynamic roles of immune cells therein. It further explores how immune dysregulation contributes to the chronicity of DFU, PUs, and VLUs, with a focus on the cellular and molecular abnormalities involved. We then highlight a range of emerging immunotherapeutic strategies, including cytokine‐based interventions, protease inhibitors, and biomaterial scaffolds engineered to modulate immune activity and support tissue regeneration. Further emphasis is placed on MSCs and their EVs, which exhibit potent immunomodulatory and proregenerative properties. These cell‐based and cell‐free therapies have shown promise in preclinical models and early‐stage clinical trials, offering novel approaches to overcome the limitations of conventional wound care. Finally, we synthesize findings from recent clinical trials evaluating immunomodulatory interventions in chronic wounds, discussing their translational potential, safety profiles, and challenges that remain on the path to clinical adoption. By integrating fundamental immunological insights with therapeutic advances, this review aims to provide a comprehensive perspective on how immunomodulation can revolutionize the management of chronic wounds and bridge the gap between bench and bedside.

## 2 Phases of Wound Healing

### 2.1 Hemostasis

The initial phase of wound healing, hemostasis, involves vascular constriction, primary hemostasis (platelet aggregation), and secondary hemostasis (fibrin clot formation via the coagulation cascade). Key players include activated platelets and fibrinogen—a liver‐derived plasma protein that is converted to fibrin to stabilize the clot. Coagulation focuses on rapidly sealing the exposed tissue to prevent fluid loss and pathogen invasion [15]. Neuronal reflex mechanisms triggered by vessel wall injury lead to rapid vasoconstriction, primarily activated by endothelin from the damaged endothelium and circulating catecholamines from injured cells. This vasoconstriction, along with platelet aggregation and plug formation (primary hemostasis), prevents excessive blood loss. Platelet activation and degranulation are critical early events in the wound healing process, playing a central role in both hemostasis and the initiation of tissue repair. The activation process begins when platelets encounter the exposed subendothelial matrix. Collagen, along with other matrix components, binds to platelet receptors such as G‐protein‐coupled receptors for thrombin. This triggers intracellular signaling pathways, essential for platelet shape change, granule release, and aggregation [16]. The coagulation cascade (secondary hemostasis) involves a series of serine proteases, with thrombin cleaving fibrinogen to form fibrin fibers. The intrinsic pathway, triggered by factor XII binding to negatively charged surfaces like collagen, plays a key role in activating this cascade. Deficiencies in the clotting process can result in prolonged bleeding [15, 16]. Platelets release adhesive glycoproteins and sphingosine‐1‐phosphate to enhance aggregation and adhesion. The platelet plug, combined with a fibrin mesh formed through the coagulation cascade, creates the thrombus and initiates signaling for wound healing. Platelet degranulation also releases various soluble effectors, including TGF‐β, platelet‐derived growth factor (PDGF), stromal cell‐derived factor 1 (SDF1/CXCL12), vascular endothelial growth factor (VEGF), and endostatin [16]. Furthermore, the innate immune inflammatory response and coagulation processes are interconnected and activate each other at the wound site, highlighting their coordinated role in the healing process [15].

In addition, deposition of angiogenesis regulators occurs at the wound site. This deposition at the wound site is a critical event during the wound healing process, facilitating the formation of new blood vessels to re‐establish oxygen and nutrient supply to the affected tissues. This process is regulated by various growth factors, cytokines, and signaling pathways that orchestrate angiogenesis, primarily through the action of endothelial cells (ECs). A key regulator in this process is VEGF, which is crucial for promoting the proliferation, migration, and differentiation of ECs into new vessel structures [17]. Studies have shown that VEGF‐A levels are significantly upregulated in healing wounds, facilitating angiogenic responses necessary for effective tissue repair [18]. In addition to VEGF, other regulatory proteins such as angiopoietin‐1 and angiopoietin‐2 also play vital roles in angiogenesis. Angiopoietin‐1 promotes blood vessel maturation and stability [19], while angiopoietin‐2 acts as a modulator, promoting vessel permeability and sprouting, especially in hypoxic conditions [20]. This interplay between angiopoietins is crucial, as studies demonstrate that their balance affects vessel remodeling and the overall angiogenic response in wounds. Moreover, fibroblasts and other MSCs in the wound microenvironment secrete angiogenic factors that further stimulate EC proliferation and new vessel formation [21].

The role of the subcutaneous fascia in hemostasis is particularly important given its anatomical positioning. Subcutaneous fascia consists of loose connective tissue that contains various cellular components, including fibroblasts, adipocytes, and vascular structures [22]. This layer acts as a mechanical barrier, protecting underlying tissues while facilitating the signaling processes necessary for hemostatic responses [23]. When injury occurs, the subcutaneous fascia becomes a site for the deposition of fibrin and other ECM components, which are instrumental in clot formation and cellular migration during the injury response. This matrix not only serves as scaffolding for cells involved in healing but also regulates local biochemical environments through the release of various cytokines and growth factors, influencing the healing trajectory [24]. This connective tissue is highly dynamic, particularly during wound healing. The traditional wound healing process involves the formation of a blood clot during hemostasis, followed by the migration and differentiation of dermal fibroblasts into myofibroblasts that form granulation tissue. However, recent studies suggest that fascia, rather than the dermal matrix, plays a pivotal role in wound closure. Fascia‐born fibroblasts is a specialized population of fibroblasts that reside in the subcutaneous fascia. These fibroblasts are distinct from other fibroblast populations found in the dermis or other tissues due to their origin and function within the fascia. These fibroblasts, particularly Engrailed‐1 lineage positive fibroblasts (EPFs), orchestrate the mobilization of fascial tissue by dragging the fascial matrix into the wound site, facilitating scar formation and wound closure. Genetic depletion of these fibroblasts leads to delayed wound healing, emphasizing their essential role in tissue repair [25].

Following a vascular injury, fascia fibroblasts are activated and migrate toward the wound site, where they begin to proliferate and synthesize large amounts of collagen and ECM proteins necessary for structural support during the healing process. This fibrogenesis assists in the formation of granulation tissue, which provides a scaffold for further cellular activities, facilitating effective wound closure [26]. Research indicates that fibroblasts not only remodel the ECM but also aid the transition from a damaged tissue state to a healed one. The process begins with the breakdown of the fibrin matrix formed during hemostasis. Fibroblasts secrete matrix metalloproteinases (MMPs) that degrade the fibrin clot and replace it with new ECM components, supporting the structural integrity of the healing tissue. The activation of signaling pathways, such as the TGF‐β/Smad pathway, is vital in regulating fibroblast proliferation and differentiation, which is crucial in modulating fibrotic responses that influence healing outcomes [25]. Further emphasizing their importance, fibroblasts also release growth factors and cytokines that attract other key immune cells to the wound site, thereby facilitating a smooth transition from hemostasis to the inflammatory phase of healing [27]. These signaling molecules orchestrate an inflammatory response necessary for combating infection and clearing debris, while also promoting vascularization and cellular migration essential for tissue repair. This dynamic interplay highlights how fibroblasts serve as both structural and regulatory cells in the wound healing environment.

### 2.2 Inflammatory Phase

As next, the inflammatory phase starts off as hemostasis halts bleeding through platelet aggregation on wound site, which usually lasts for 2 days. Immune cells such as neutrophils promote angiogenesis and re‐epithelialization by releasing cytokines (e.g., TNFα, IL‐1β, IL‐1Rα, IL‐12, IL‐17A, and VEGF) and chemokines (e.g., CXCL1, CXCL8, CXCL9, CXCL10, CCL3, and CCL4), which cleanse the infection and recruit and activate skin regenerating cells e.g. fibroblasts and epithelial cells [28]. The inflammatory phase of wound healing begins with the activation of transcription‐independent pathways triggered by trauma, including Ca^2+^ waves, ROS, and purinergic signaling [29]. Within minutes of injury, Ca^2+^ concentrations increase at the wound edges and spread toward the center, signaling the release of damage‐associated molecular patterns (DAMPs), hydrogen peroxides (H_2_O_2_), lipid mediators, and chemokines, which are essential for neutrophil recruitment [30, 31]. H_2_O_2_ plays a critical role in minimizing infection by further attracting neutrophils, activating keratinocyte regeneration, and promoting new vessel formation [30].

The inflammatory phase can be divided into early and late phases. The early phase is primarily defined by neutrophil activity, which involves recruitment, rolling, adherence, and diapedesis through tissues to the wound site. Neutrophils make up about 50% of cells at the wound site within the first day and are attracted by “find me” signals such as DAMPs, H_2_O_2_, and chemo‐attractants (e.g., CXCL4, CXCL8, CXCL10, CXCL12, CCL3–5). These signals create a gradient that guides neutrophils to the wound, where they form a provisional barrier to prevent microbial invasion. Additionally, granulocyte colony‐stimulating factor (G‐CSF) and CXC chemokines are released, promoting the entry of mature neutrophils from the bone marrow to the wound site. Neutrophil migration is initially mediated by CXCR2 signaling along the vessel endothelium and chemokine gradients. Later, formyl‐peptide receptor‐dependent signaling directs neutrophils into necrotic zones through necrotaxis, guided by formyl‐peptide gradients. This process ensures the efficient clearance of debris, foreign material, and bacteria, which is critical for continued wound healing.

Neutrophils combat infection by releasing toxic granules (e.g., lysozyme, cathepsin G, elastase, proteases) [32], generating an oxidative burst, initiating phagocytosis, and producing neutrophil extracellular traps (NETs). NETs are chromatin filaments coated with histones and proteases that trap and degrade pathogens through NETosis [33]. NETosis occurs in two forms: suicidal late NETosis, where ROS‐induced neutrophil death releases NETs, and early vital NETosis, where neutrophils remain viable and continue participating in phagocytosis and chemotaxis [33]. While NETs help clear infections, impaired NET production can hinder wound healing, increasing wound infection and nonhealing. As the infection resolves, neutrophil activity shifts, and neutrophils are eliminated by extrusion and apoptosis, contributing to wound slough [32, 33]. Macrophages play a critical role in subsequent healing phases, with their depletion causing delays and their increase promoting faster wound healing, especially in diabetic wounds. Macrophages initiate the late inflammatory phase of wound healing by phagocytosing cellular debris [34]. Their number peaks around day 3 and decreases by day 10. Macrophages differentiate from monocytes and produce signaling molecules that activate keratinocytes, fibroblasts, and ECs [35]. Initially, M1 macrophages are proinflammatory, phagocytosing pathogens and secreting proangiogenic cytokines like VEGF to promote angiogenesis [36]. As inflammation subsides, M1 macrophages transit to the M2 form, aiding vessel formation and remodeling via vascular mimicry and Tie2 expression [37]. Macrophages can recruit more macrophages through the release of chemoattractants such as monocyte chemoattractant protein 1 (MCP‐1) [38]. T lymphocytes, attracted by IL‐1, especially dendritic epidermal T‐cells (DETCs), migrate into the wound. DETCs change morphology and release keratinocyte growth factors, promoting keratinocyte proliferation and initiating epithelialization. Depletion studies have shown that DETC depletion delays wound closure [39, 40].

Fibroblasts derived from the fascia have been observed to adopt a proinflammatory phenotype after injury, aiding in the rapid recruitment of immune cells such as macrophages and neutrophils to the wound site [41]. These specialized fibroblasts express various cytokines and chemokines. For instance, they promote the secretion of IL‐6, a key proinflammatory cytokine that plays a role in the activation and differentiation of T and natural killer (NK) cells, thereby enhancing the immune response to injury [42]. The presence of these inflammatory mediators not only signals for immune cell recruitment but also regulates the local inflammatory environment, ensuring an adequate response to potential pathogens. Moreover, fibroblasts exert influence over macrophage polarization, often shifting macrophages from a M1 phenotype to a reparative M2 phenotype, which is crucial for the transition toward the proliferative phase of healing [43]. This modulation helps prevent excessive inflammation that could lead to chronic wounds. Additionally, studies highlight the significant relationship between fibroblasts and various inflammatory signaling pathways. Increased expression of VEGF by fascia‐derived fibroblasts has been linked to enhanced angiogenesis during inflammation, which aids in delivering more immune cells to the site of injury and supporting tissue repair [44]. The persistent action of fibroblasts in modulating these signals is essential for regulating inflammation duration and intensity, ensuring a swift transition to subsequent healing phases.

### 2.3 Proliferative Phase

The proliferation phase marks the onset of tissue repair, often referred to as the granulation phase due to the granular appearance of newly formed tissue in histological and macroscopic views. This phase is characterized by the formation of new stroma by fibroblasts, the sprouting of new blood vessels, and the deposition of ECM, including collagen synthesis [45]. Fibroblasts from the subcutaneous fascia are the primary effector cells in this phase, producing ECM components, particularly collagen. Studies demonstrate that during this phase, there is an increase in fibroblast numbers, especially in the first week postinjury, as these cells migrate into the wound site to synthesize and organize collagen fibers into a scaffold [46]. Furthermore, fibroblasts in the fascia are influenced by signaling molecules that modulate their behavior during the proliferative phase. For instance, transforming TGF‐β is crucial for stimulating fibroblast proliferation and collagen synthesis. TGF‐β signaling facilitates the differentiation of fibroblasts into contractile myofibroblasts, which actively contribute to wound contraction and tissue remodeling [45]. This transition is essential for minimizing wound size and restoring tissue integrity. A key aspect of the proliferation phase is the restoration of the vascular system, which not only aid in initial hemostasis, blood loss reduction, and establishes a provisional wound matrix but also provide blood clot‐derived cytokines and growth factors. This creates a provisional wound microenvironment consisting of essential cytokines and growth factors acting as a starting point for new vessel formation and the restoration of blood flow, which is crucial for delivering oxygen, nutrients, and sustaining cell metabolism necessary for healing [47].

Angiogenesis, the process of new vessel formation, can be subdivided into two phases: the proangiogenic phase, marked by excessive blood vessel production, and the antiangiogenic phase, where the vascular network matures and the number of vessels decreases [47]. Microvascular ECs and, to a lesser extent, pericytes play a main role in angiogenesis [48]. The process begins with the detection of low oxygen levels, which activate ECs via hypoxia‐responsive growth factors like VEGF and PDGF [49]. Capillary basement membrane degradation by enzymes allows ECs to proliferate. Tip cells, the leading ECs in the sprouting process, extend filopodia to navigate the ECM along a proangiogenic gradient, guided by VEGF‐A receptors. Tip cells secrete proteolytic enzymes to create a path for the sprouting capillary. These cells sense VEGF concentrations and align with the highest gradient, while stalk cells follow and elongate the sprout, forming the trunk of the new capillary. Once sprouting tip cells converge, they fuse to form tubules that connect to existing vessels, re‐establishing blood flow. However, these vessels remain leaky, allowing immune cell infiltration until blood flow is fully restored. Vascular maturation begins when tissues receive adequate oxygen, causing VEGF concentrations to decrease. This triggers the recruitment of pericytes to the outer vascular endothelium, deposition of ECM, and the application of shear stress, leading to the stabilization of the new capillaries [49].

VEGF plays a crucial role not only in EC activation but also in fibroblast function, which can further promote angiogenesis in wounds. For instance, recent studies have shown that overexpressing VEGF in fibroblasts enhances angiogenesis and accelerates granulation tissue formation during the early phases of wound healing [50]. The formation and maturation of vessels during wound healing do not follow a straightforward pattern. Initially, a ring of irregularly organized vessels is formed at the wound edge, exhibiting inconsistent blood flow. As healing progresses, this vascular ring contracts toward the center of the wound, leaving behind radially oriented vessels that supply the wound and connect with the uninjured skin. These vessels are more organized and demonstrate near‐physiological blood flow [49]. Any disturbances in this neovascularization process can lead to complications, such as chronic ulcers, which are commonly observed in conditions like venous insufficiency, arteriosclerotic diseases, and DFUs.

### 2.4 Re‐Epithelialization Phase

The epidermis consists of keratinocytes connected by cell–cell junctions, primarily desmosomes, with a specialized ECM called the basement membrane linking the basal layer of the epidermis to the dermis via hemidesmosomes and focal adhesions [51]. Cell motility, driven by mechanisms of protrusion, adhesion, and traction, is crucial for the epithelialization process, which begins hours after injury. During this phase, keratinocytes transform from stationary, cobblestone‐like cells to flat, migrating cells. This transformation is known as epithelial–mesenchymal transition (EMT), specifically a type II EMT, which occurs during tissue repair [52]. EMT involves the change from an adherent epithelial morphology to a more motile mesenchymal phenotype. During migration, keratinocytes engage in lamellipodial crawling and shuffling across the wound site. To achieve this, they must loosen their cell–cell and cell–substratum contacts, which are maintained by desmosomes and hemidesmosomes, and rearrange their actin cytoskeleton and adhesive structures. The apical–basal polarity of the cells is altered, allowing leading‐edge keratinocytes to migrate laterally across the wound, a critical step in restoring the epidermal layer [52, 53]. E‐cadherin plays a key role in maintaining cell adhesion and immobility, while vimentin upregulation leads to the downregulation of E‐cadherin and the transition to a mesenchymal phenotype with corresponding markers. Additionally, integrin expression is temporally upregulated to further increase cell motility [52, 54]. Interestingly, EMT does not always fully occur; cells may exhibit a hybrid phenotype with both epithelial and mesenchymal characteristics along a gradient [55].

Behind the leading keratinocytes, the “second row” of activated keratinocytes proliferates to replenish the cell pool. The leading keratinocytes migrate over the wound site, utilizing fibrin, fibronectin, and vitronectin from the blood clot as substrates for lamellipodial crawling [56]. Rather than migrating centripetally to the wound center, these cells change shape, break cell–cell contacts, and rearrange themselves at the anterior margin, a process known as shuffling [56]. Once the cells reach the wound center, contact inhibition halts their migration, completing the wound coverage. While this process is typical in human wound healing, rodents primarily rely on wound contraction as the main mechanism for wound closure, which should be considered when interpreting experimental results from these models [57]. In aged skin, wound healing, particularly the formation of a new epidermis, is significantly slower. This is attributed to the reduced activity of eccrine sweat glands, which, although present in the same density as in young skin, have diminished activity in forming epithelial outgrowths during skin repair by 50% in aged skin [58]. This reduction leads to weakened cell–cell contacts, increased intercellular gaps, fewer desmosomes, thinner epidermal repair, and overall delayed wound closure in aged individuals [58].

## 3 Immune Regulation of Acute Wound Healing

Immune regulation plays a crucial role in wound healing, coordinating various cellular activities to ensure effective tissue repair and regeneration. The process is complex, involving a variety of immune cells, cytokines, and growth factors that work together to manage inflammation, promote healing, and restore tissue integrity. Immune cells are pivotal in development and healing of acute and chronic wounds through inflammatory reaction. Although, adequate inflammation is necessary to fight infections for preventing tissue death through septicemia, prolonged inflammation can interfere with the normal healing of wounds, causing scar formation on the healed tissue [59]. On the basis of particular roles of inflammatory cells in scar formation, several therapeutic strategies were developed aiming at specific immune targets to induce immunomodulation to enhance healing of chronic injury and skin regeneration [60].

In an acute wound, the first blood cells to arrive on the injury site are platelets (thrombocytes) for blood coagulation. They also interact with exposed collagen and damaged ECM which trigger secretion of growth factors including regulatory cytokines such as TGF‐β which regulates proliferation, differentiation and survival of lymphocytes [61]. These growth factors attract immune cells such as neutrophils, mast cells, and macrophages from nearby tissue to the wound site [62]. The immune cells produce then proinflammatory mediators like interleukin‐1 (IL‐1), TNF‐α, and interferon gamma (IFN‐γ), as well as a variety of growth factors like epidermal growth factor (EGF), and insulin‐like growth factor 1 (IGF‐1), which are important mediators of the tissue repair [63]. Immune cells involved in the wound healing process are enlisted in Table 1.

**TABLE 1:** Immune cells involved in the wound healing process: Sources, timing of recruitment, and their functional roles.

Immune cell type	Source & timing	Functional role	References
Neutrophils	Bone marrow‐derived; enter wound sites early. First responders (within hours postinjury)	First responders to injury; remove pathogens and debris, contributing to the inflammatory phase; may delay healing if excessive	[4, 32, 33, 64, 65]
Macrophages	Originates from monocytes that migrate from the bloodstream. Arrive shortly after neutrophils (1–3 days)	Essential in phagocytosis and transitioning from inflammation (M1) to healing (M2) states; secrete growth factors and cytokines that support tissue repair	[6, 34, 35, 37]
Dendritic cells	Derived from bone marrow; present in tissues. Arrive within 2–4 days	Act as antigen‐presenting cells that activate T‐cells and promote adaptive immune responses; crucial for coordinating inflammation and healing	[40]
T‐cells	Generated in the thymus; enter via circulation. Presence peak around 3–7 days	Regulate immune responses; CD4+ T helper cells support macrophage activation and cytokine production, promoting inflammation and tissue repair	[39, 66]
B cells	Developed in bone marrow; migrate to tissues. Present around 1 week after injury	Produce antibodies and cytokines that can modulate inflammation, contributing to tissue repair; their absence can affect wound healing dynamics	[67, 68]
Mast cells	Generated in bone marrow; resident in tissues. Present throughout but activated within days	Release inflammatory mediators such as histamine; participate in recruiting other immune cells to the wound site, influencing blood flow and healing	[69, 70]
Natural killer cells	Produced in the bone marrow; enter the wound site via circulation. Present within the first few days	Contribute to immune surveillance and clearance of infected or damaged cells; support angiogenesis and tissue remodeling during healing	[42, 71]

Neutrophils are amongst the first immune cells at the wound site with the primary function of wound sterilization through phagocytosis of damaged tissue and infectious particles [32, 64]. They secrete complex antimicrobial agents such as proteases, peptides, and ROS, which limit virulent pathogen invasion in wounded tissue and commit microbial clearance through NET formation [72]. Once the injury site is disinfected, neutrophils secrete cytokines and growth factors, for example, VEGF, to promote proliferation of nearby fibroblasts, keratinocytes, and ECs [50]. Neutrophils also do chemotaxis of inflammatory cells and remain on the wound site for about 24 h before undergoing apoptosis [72, 73].

As neutrophils undergo apoptosis, they are subsequently engulfed by macrophages in a process known as efferocytosis [74, 75]. This clearance is critical as it not only prevents prolonged inflammation but also releases signals that activate macrophages to transition from a M1 phenotype to a reparative M2 phenotype, promoting tissue regeneration [74]. Tissue‐resident macrophages and monocyte‐derived macrophages play critical roles in wound healing through distinct but complementary mechanisms [34]. Tissue‐resident macrophages, derived from embryonic yolk sac progenitors, are strategically located in the skin and other tissues. These macrophages exhibit plasticity; they can switch from a M1 state to an anti‐inflammatory M2 state, enhancing tissue repair and promoting angiogenesis as healing progresses. Their role in maintaining tissue homeostasis is vital, as they release cytokines and growth factors that orchestrate the healing process and promote the resolution of inflammation [34, 35]. In contrast, macrophages originate from circulating monocytes that migrate to the wound site in response to inflammatory signals [76]. These macrophages typically arrive within the first few days after injury, where they initially take on an M1 phenotype that aids in pathogen clearance and inflammation. As wound healing shifts from inflammation to tissue regeneration, these macrophages can switch to an M2 phenotype, which is essential for repair processes such as collagen deposition and granulation tissue formation. The interplay between tissue‐resident and monocyte‐derived macrophages ensures that the wound bed has a robust immune response during the early inflammatory phase, followed by a coordinated effort for tissue remodeling and healing [76, 77]. T‐cells, particularly T regulatory cells (Tregs), also play a significant role in modulating the immune response during wound healing [78]. Recent studies have highlighted that Treg cells can promote the transition of macrophages from a proinflammatory to a repair‐focused state, thus enhancing tissue regeneration. They assist in controlling inflammation and ensuring that the reparative processes initiate promptly, underscoring the interplay between adaptive and innate immunity [78, 79].

Understanding the dynamics of these macrophage populations is crucial, especially given their implication in diabetic wound healing, where aberrant macrophage activity can lead to chronic inflammation and delayed repair. Overall, the coordinated actions of both tissue‐resident and monocyte‐derived macrophages establish a finely tuned balance between inflammation and healing, which is necessary for effective tissue regeneration. As inflammatory macrophages ingest the neutrophils on local sites, macrophages are a crucial component of the auto‐regulatory loop involved in processing inflammation [65].

Macrophages are believed to have a major role in inflammation, phagocytosis, and wound healing as they are crucial mediators in subtle transitions between the four stages of wound healing. In particular, skin wound models demonstrated a variety of these functions, including scavenging, phagocytosis, antigen presentation during the inflammatory phase [76], stem cell recruitment and revascularization all through the proliferative phase [80], and extracellular signaling transduction throughout the remodeling phase [81]. Recently, research has focused on different macrophage subtypes and their potential to minimize inflammation and advance tissue regeneration. M1 macrophages differentiated from monocytes by exposure to IFN‐γ and TNF‐α [82]. M1 macrophages are also induced by T‐cells secreted cytokines, recognition of pathogen‐associated molecular patterns, such as bacterial lipopolysaccharides (LPSs) and peptidoglycan, or DAMPs, such as released intracellular proteins and nucleic acids [30]. M1 produce proinflammatory cytokines such as IL‐1β, IL‐6, TNF‐α, and IFN‐γ [37].

In contrast, a range of stimuli, including IL‐1β, IL‐10, TGF‐β, IL‐4/IL‐13, Toll‐like receptor (TLR) ligands, and glucocorticoids, can cause induction of M2 macrophages. M2 macrophages comprise of several subgroups defined by their activation stimuli and functional characteristics. These macrophages are generally categorized into four subsets: M2a, M2b, M2c, and M2d, each responding to different cytokines and environmental cues. M2a macrophages, for instance, are stimulated by ILs such as IL‐4 and IL‐13, resulting in the production of anti‐inflammatory cytokines such as IL‐10, TGF‐β, CCL17, CCL18, and CCL22, and promoting tissue repair processes like angiogenesis and collagen deposition. M2b macrophages respond to immune complexes or LPSs, and release both pro‐ and anti‐inflammatory cytokines such as TNF‐α, IL‐1, IL‐6, and IL‐10. M2c macrophages, activated by IL‐10 or glucocorticoids, perform critical roles in the phagocytosis of apoptotic cells and also release IL‐10, TGF‐β, CCL16, and CCL18, for the resolution of inflammation and promoting ECM remodeling. M2d macrophages arise from the stimulation of monocytes in response to specific cytokines such as IL‐4, IL‐13, and IL‐10, and, in turn, secrete VEGF, which aid in angiogenesis [37, 83].

Traditional understanding of macrophage polarization has primarily centered around the dichotomy between M1 and M2 macrophages. M1 macrophages are commonly considered proinflammatory, produce inflammatory cytokines, ROS, and proteases, to combat pathogens [37]. Conversely, M2 macrophages are associated with anti‐inflammatory functions and tissue repair processes, promoting wound healing through secretions of growth factors and cytokines that facilitate collagen deposition and angiogenesis. Recent studies utilizing single‐cell RNA sequencing (scRNA‐seq) techniques have significantly illuminated the complexities of macrophage subsets involved in wound healing, revealing their diverse roles during the regeneration process. These insights allow researchers to dissect the heterogeneity of macrophages, uncovering specific gene expression profiles associated with distinct macrophage subpopulations. A comprehensive study utilized scRNA‐seq to map the cellular landscape of human skin wound healing over time [84]. Researchers identified proinflammatory macrophages which support re‐epithelialization in the inflammatory phase. They identified 11 distinct myeloid cell populations in acute wounds, including four macrophage clusters: Mac_inf (APOE+ and CXCL1+), Mac1 (IL1B+, THBS1+, and EREG+), Mac2 (DAB2+ and C1QA/B+), and Mac3 (MMP19+, MMP9+, and VEGFA+); four dendritic cell (DC) clusters: plasmacytoid DC (pDC, ACOT7+, LTB+, and IGKC+), conventional DC1 (cDC1, CLEC9A+, and WDFY4), cDC2 (CD1C+, IL1R2+, and CLEC10A+), and DC3 (CCR7+ and LAMP3+); and Langerhans cells (LCs) (CD207+ and CD1A+). In the early stages, proinflammatory macrophages (Mac_inf and Mac1) transiently increase, characterized by the upregulation of HIF1α and proinflammatory cytokines such as TNF‐α, IL‐1β, and CCL2. Conversely, proresolution macrophage markers (MRC1, IL‐10, TGF‐β, and PDGFB) are initially downregulated. This dynamic transition is crucial for proper wound healing, and its disruption may contribute to chronic wound conditions, highlighting potential therapeutic targets [84].

In another study, researchers investigated the dynamics of macrophage subtypes during skin wound healing in both mice and humans [85]. They identified two primary macrophage subtypes. M1 macrophages (proinflammatory) are characterized by the expression of markers such as inducible nitric oxide (NO) synthase (iNOS). In the study, M1 macrophages were found to be more abundant in the early stages of wound healing. Specifically, in mouse wound specimens, F4/80⁺CD80⁺ M1 macrophages were predominant on day 3 postinjury. Similarly, in human wound specimens, CD68⁺iNOS⁺ M1 macrophages were more prevalent in wounds aged 2–5 days. M2 macrophages, marked by the expression of CD163, became more prominent during the later stages of wound healing. In mice, F4/80⁺CD206⁺ M2 macrophages were notably detected in wounds at day 6. In human samples, CD68⁺CD163⁺ M2 macrophages increased in wounds older than 5 days. The study highlighted that the ratio of M1 to M2 macrophages (M1/M2 ratio) can serve as a potential marker for determining wound age. For instance, an M1/M2 ratio greater than 2.5 was indicative of wounds aged 2–5 days. These findings suggest that monitoring the balance between M1 and M2 macrophages can provide valuable insights into the wound healing process and may have practical applications in forensic science for estimating wound age [85].

One comprehensive study by Richards et al. [86] aimed to identify marker genes indicative of specific wound subregions, such as the wound bed, wound border, and peri‐wound areas. By employing scRNA‐seq combined with machine learning algorithms, the findings delineated significant differences in gene expression associated with macrophage populations across these different wound areas, highlighting the importance of localized macrophage activity in response to varying wound microenvironments. This suggests that macrophages do not function uniformly across the entire wound but instead adapt their phenotype based on localized microenvironmental cues. The study reveals that macrophages within chronic wounds exhibit distinct gene expression patterns depending on the wound type (e.g., diabetic ulcers vs. VLUs) and the specific subregions within the wound. RNA seq studies also revealed wound type‐specific macrophage profiles. Diabetic ulcers showed an overrepresentation of inflammatory macrophages with a failure to resolve inflammation, VLUs displayed macrophage profiles associated with excessive tissue degradation, likely due to high levels of MMPs and PUs demonstrate a dysregulated macrophage response with inefficient clearance of apoptotic cells and ECM turnover issues [86]. The findings emphasize the need for personalized macrophage‐targeted therapies to restore a balanced inflammatory response and promote wound healing.

In a notable study by Dube et al. [87], age‐related alterations in macrophage distribution and function were examined, revealing that aged macrophages express distinct inflammatory profiles compared with their younger counterparts. Specifically, their scRNA‐seq analysis identified increased M1 macrophage prevalence, which correlates with delayed healing in cutaneous wounds. The findings highlighted that the aberrantly heightened inflammatory responses in aged macrophages may impair wound recovery, suggesting that interventions aimed at modulating macrophage activation could improve healing outcomes. This emphasizes the importance of understanding macrophage subtypes and their functional implications in chronic wound healing processes [87]. Another relevant contribution comes from Vu et al. [88], who explored cellular composition and communication changes in aged skin wounds through scRNA‐seq. They identified distinct macrophage populations exhibiting alterations in gene expressions, such as cytokines involved in inflammation, thereby influencing wound healing dynamics. Their findings suggest that the mechanical environment and cellular interactions significantly shape macrophage behavior during the healing process, further emphasizing the importance of targeted interventions in chronic wound scenarios [88].

## 4 Immune Dysregulation of Chronic Wound Healing

Chronic wounds are identified as those that do not exhibit signs of healing within 4–12 weeks [89]. Impaired cellular function, exudation, recurrent infection, persistent inflammation, tissue necrosis, inadequate re‐epithelialization, reduced angiogenesis, excessive protease activity, and excessive ROS generation are typical characteristics of nonhealing wounds [89, 90, 91]. These wounds often reflect an underlying immune dysregulation that hampers effective healing. The persistence of inflammation and a failure to transition through the normal phases of healing—namely hemostasis, inflammation, proliferation, and reepithelialization/remodeling—characterize these wounds, leading to a prolonged inflammatory state. Several studies have explored various aspects of immune dysregulation in chronic wounds, emphasizing the contributions of specific immune cell populations, particularly macrophages, in this process. A significant contributor to chronic wound pathology is the persistent presence of M1 macrophages, which secrete proinflammatory cytokines and perpetuate an inflammatory microenvironment. For instance, Xiong et al. [92] reported that chronic wounds contain an overwhelming majority of M1 macrophages, which can comprise approximately 80% of the immune cell population at the edges of chronic wounds. This imbalance disrupts the normal shift to M2 macrophages, which are essential for promoting tissue repair and resolution of inflammation [92]. This failure to switch from a proinflammatory to a reparative macrophage phenotype significantly impairs the healing process, leading to tissue necrosis and chronicity. Chronic wounds, such as PUs, VLUs, and DLUs, often exhibit severe immune dysregulation. Understanding the unique immune profiles associated with these types of chronic wounds can inform better therapeutic strategies.

### 4.1 Immune Dysregulation in PUs

PUs, commonly known as bedsores, develop due to prolonged pressure on the skin, leading to tissue ischemia and necrosis. The development of PUs involves a complex interplay of mechanical, biological, and chemical factors, encountered in patients with limited mobility. A significant factor contributing to immune dysregulation in PUs is the overwhelming presence of proinflammatory macrophages. Arai et al. [93] highlighted that the healing of PUs is significantly delayed in patients with comorbidities such as cardiovascular disease, which leads to peripheral circulatory disturbances and prolonged inflammation. The persistent recruitment of M1 macrophages—characterized by their proinflammatory profile—leads to the excessive production of cytokines such as TNF‐α and IL‐6. These cytokines contribute to a sustained inflammatory environment that interferes with the wound healing process [93]. Moreover, Guo et al. [94] identified that PUs exhibit dysregulated expression of cyclooxygenase‐2 (PTGS2), associated with the inflammatory response in these lesions. Their study noted that targeting PTGS2 with therapeutic compounds could reduce inflammation, promote angiogenesis, and enhance healing outcomes in pressure sore management [94]. This underscores the concept that modulation of local inflammatory responses can influence healing efficacy.

The transition from inflammation to repair is often disrupted in PUs due to continuous inflammation. According to Álvarez‐Viejo et al. [95], chronic pressure injuries develop when the natural repair mechanisms fail, causing an accumulation of inflammatory cells that prevent effective remodeling and epithelial proliferation. They emphasized that without proper regulation, wounds will be caught in a state of chronic inflammation characterized by fibrosis and persistent infiltration of inflammatory cells. Furthermore, Dabas et al. [96] reported that the chronic nature of PUs is associated with excessive protease activity and insufficient angiogenesis, further complicating the healing landscape [96]. This persistent inflammation not only leads to elevated levels of MMPs, which degrade the ECM, but also impedes cellular migration and proliferation necessary for wound healing. Research by Wickström et al. [97] highlighted the clinical ramifications of immune dysregulation in PUs, articulating that chronic inflammation often leads to increased hospitalization rates and associated healthcare costs [97]. The presence of wounds that do not progress through the normal healing stages presents a considerable burden, not only on patients but also on healthcare systems.

The role of the microbiome in modulating immune responses in chronic wounds, including PUs, is increasingly recognized. Verbanic et al. [98] noted that the microbiome diversity in chronic wounds, including PUs, is significantly altered, contributing to dysregulated immune responses and impairing healing [98]. An imbalance in microbial communities can exacerbate local inflammation and predispose wounds to infections, necessitating an integrated approach to wound management that considers both immune factors and microbial influences. Researchers further emphasized that the persistence of inflammatory infiltrates, especially M1 macrophages, correlates with the presence of biofilms in chronic wounds. These biofilms can harbor pathogenic bacteria that resist clearance by the immune system, perpetuating a cycle of inflammation and tissue damage [99].

Moreover, researchers conducted a comprehensive proteomic analysis of wound fluid from 42 PUs in 32 subjects over a 6‐week period, collecting samples from both the interior and periphery of the wound beds [100]. They found differential protein expression as the study identified 21 proteins that significantly distinguished healed wounds from chronic, nonhealing ones. They also reported 19 proteins that exhibited differential expression between the interior and periphery of the wounds. Lower levels of pyruvate kinase isozymes M1/M2, profilin‐1, Ig lambda‐1 chain C region, and Ig gamma‐1 chain C region in the periphery suggest metabolic and immune deficiencies in chronic wounds. A reduction in glycolysis (pyruvate kinase M1/M2) and immune‐related proteins (Ig chains) indicates impaired cellular energy metabolism and a weakened immune response, which are characteristics of nonhealing wounds. Higher levels of KRT6A, KRT14, S100A7, alpha‐1‐antitrypsin precursor, hemoglobin subunit alpha, and hemoglobin subunit beta in the periphery reflect an increased but ineffective inflammatory response and altered tissue remodeling. While keratins (KRT6A, KRT14) are involved in epithelial repair, their accumulation in chronic wounds may indicate stalled re‐epithelialization. Similarly, S100A7 is linked to prolonged inflammation, and the presence of hemoglobin subunits suggests oxidative stress rather than effective oxygen delivery for healing. Overall, these proteomic patterns highlight the dysregulated metabolic, immune, and tissue repair processes in chronic PUs, contributing to delayed healing [100].

Furthermore, researchers conducted a comparative single‐cell transcriptomic analysis of epidermal cells from PU wound edges, uninjured skin, and acute wounds in healthy donors [101]. They reported that the upregulation of MHC class II expression in keratinocytes, driven by IFN‐γ in the wound environment, may contribute to impaired immune responses and hindered healing in chronic PUs [101]. Therefore, the identification of immune dysregulation of PUs provides critical insights for therapeutic interventions. Incorporating strategies aimed at modulating the immune response, such as the use of anti‐inflammatory agents or agents that promote M2 macrophage polarization, may have profound implications for improving the management of PUs.

### 4.2 Immune Dysregulation in Diabetic Ulcers

DLUs are a major complication associated with diabetes mellitus, where restricted blood circulation and nerve damage can lead to the development of nonhealing wounds on the feet, leading to severe morbidity and even amputation. Central to the chronic nature of diabetic ulcers is immune dysregulation, involving a complex interplay of inflammatory and reparative processes that are severely disrupted. All four wound healing phases are impacted by diabetes mellitus [102]. Due to the increase of inflammatory cytokines like TNF‐α and the decreased production of prohealing mediators like IL‐10 and TGF‐β, DLU present a significantly proinflammatory profile. This results in the activation and degranulation of CD8+ T‐cells, macrophage polarization toward the M1 phenotype, and tissue necrosis [103]. Yi et al. [104] describe how dysregulated immune cells, particularly M1 macrophages, play a crucial role in perpetuating inflammation in DLUs. The presence of M1 macrophages disrupts the normal polarization to M2 macrophages, which are essential for tissue repair and resolution of inflammation. This continued state of inflammation not only hinders healing but also creates an environment conducive to further ulceration [104]. Myeloid cell populations, like macrophages, monocytes, and neutrophils, which are present for a long time throughout the late stages of inflammation, are indicative of chronic wound healing. However, throughout the procedure, the proportion of LCs, dermal DCs and eosinophils decreases [90, 105]. Mast cells play a role in the onset of chronic wounds as well. For instance, in DLUs, cutaneous mast cells degranulate, and the activity of these cells is downregulated, which facilitates wound healing [106].

Other than macrophages, the dysregulation of T‐cells, microRNAs (miRNAs), cytokines, chemokines, growth factors, and MMPs contributes significantly to the pathophysiology of DLUs, impairing the healing process and increasing susceptibility to further complications. T‐cells are involved in keeping nonhealing wounds in a proinflammatory phase. Recent studies have emphasized that in diabetic conditions, T‐cells exhibit altered activation states. For instance, researchers demonstrated that DLUs often contain a higher proportion of proinflammatory Th1 cells compared with Th2 cells, which shifts the immune response toward persistent inflammation rather than facilitating the reparative processes necessary for healing. Furthermore, inflammatory T‐cells subtypes including Th1, Th17, and Th22 are more prevalent in DLU patients [107, 108]. Cheng et al. [109] investigated the immunological factors contributing to DFU healing. Researchers identified 217 differentially expressed genes (DEGs) between ulcerated and healthy skin and 37 DEGs between healing and nonhealing ulcers. scRNA sequencing and flow cytometry analyses indicated a significant reduction in CD8+ T‐cells in ulcerated skin, whereas healing ulcers showed increased levels of CD8+ T‐cells, B cells, and NK cells, suggesting a critical role for these immune cells in wound recovery. Additionally, RT‐qPCR confirmed the involvement of key CD8+ T‐cells‐related genes in DFU healing [109]. These findings highlight the potential immunological resilience mechanisms in DFUs, emphasizing CD8+ T‐cells as a crucial factor in wound healing, hence providing new insights into potential therapeutic targets for DFU treatment. Additionally, uncontrolled hyperglycemia has been shown to impair T‐cells function, reducing their ability to promote wound healing effectively [110]. Moreover, a study by Dong et al. [111] identified a significant alteration in the immune cell landscape in the DFU microenvironment using scRNA‐seq, particularly in the inflammatory response. The analysis revealed a M1 phenotype, with overexpression of genes involved in inflammatory pathways like nuclear factor kappa B (NF‐κB) and TNF signaling. This sustained inflammatory state is further exacerbated by increased proportions of CD8+ T‐cells with inflammatory gene signatures, which contribute to tissue damage and delayed healing. Additionally, elevated levels of proinflammatory cytokines and chemokines, such as TNF‐α, IL‐1β, and IL‐6, were observed, perpetuating the inflammatory response and preventing the transition to the resolution phase of healing.

Furthermore, the development of chronic wounds is controlled epigenetically by miRNAs, critical posttranscriptional regulators of gene expression that influence various cellular processes in wound healing. Macrophages secrete proinflammatory factors such as IL‐1β, IL‐6, and TNF‐α through pathways like NF‐κB and TNF, which in turn regulate the expression of specific miRNAs. These miRNAs can either amplify or suppress inflammation by targeting transcription factors or signaling molecules. An imbalance in miRNAs regulating macrophage polarization, particularly in DFUs, contributes to impaired healing. miRNAs govern inflammatory responses by modulating signaling pathways. miRNAs control several signaling pathways such as VEGF, PI3K/Akt/mTOR, Wnt/β‐catenin, TGF‐β/Smad, and NF‐κB pathways during the healing of chronic wounds [112, 113]. Recent research has identified specific miRNAs that modulate inflammation and repair mechanisms in DLUs. miR‐132, miR‐203, miR‐23a, b and c, miR‐145, miR‐29b and c, miR‐126, miR‐503, and miR‐34a are associated with diabetic foot [114]. High glucose levels promote M1 polarization and the secretion of miR‐503, which inhibits IGF‐1R expression, thereby reducing EC viability, migration and tube formation, ultimately delaying wound healing [114].

In contrast, miR‐132 downregulates key inflammatory pathways (TLRs, IL‐1 receptor‐associated kinase 1 [IRAK1], and TNF receptor‐associated factor 6 [TRAF 6], NF‐κB, and nonobese diabetic [NOD]‐like receptors) in monocytes, macrophages, and keratinocytes [112, 113]. Notably, diabetic wounds exhibit lower miR‐132 expression than normally healing wounds [113]. However, local administration of miR‐132 mimics in diabetic mice significantly accelerated wound closure, enhanced keratinocyte proliferation at wound edges, and reduced inflammation. Furthermore, delivering miR‐132 mimics in liposomes mixed with pluronic F‐127 gel improved re‐epithelialization in human skin wounds, highlighting its therapeutic potential for DFU treatment [112]. However, miR‐132‐3p has been implicated in the pathophysiology of neuropathy associated with DFUs. Studies have shown that patients with peripheral neuropathy exhibit a 2.6‐fold increase in miR‐132‐3p expression in white blood cells compared with healthy controls. Additionally, sural nerve biopsies from neuropathy patients with pain displayed a slight upregulation of miR‐132‐3p compared with those without pain. In animal models, elevated spinal levels of miR‐132‐3p were observed following nerve injury, correlating with persistent pain behaviors. Pharmacological inhibition of miR‐132‐3p in these models alleviated pain, suggesting its pronociceptive role in neuropathic pain conditions. These findings indicate that increased miR‐132‐3p expression may contribute to neuropathic pain mechanisms in DFU patients [114]. Researchers provided evidence that altered expression of miR‐146a [115] and miR‐155 [116] in DLUs correlates with chronic inflammation. These studies also reported that there can be a potential link between diminished miR‐146a expression and heightened oxidative and ER stress in more severe grades of DFUs [115]. This suggests that these miRNAs could be targeted to restore normal immune function and promote healing.

During the healing process, immune and other cells release and control cytokines, chemokines, and growth factors. Recent studies revealed that DFU patients had significantly higher serum levels of proinflammatory cytokines, including TNF‐α, G‐CSF, growth‐related oncogene (GRO), MCP‐1, and leptin compared with nonulcerated diabetic patients [113]. Additionally, the study found elevated levels of PDGF‐AA and fibroblast growth factor‐2 (FGF‐2) in DFU patients, particularly those with nonhealing ulcers, indicating a resistance to growth factor action in these individuals [113]. Furthermore, increased expression of MMP‐9 was observed, which contributes to ECM degradation and impedes proper wound healing. Overall, the study concluded that the heightened inflammation, elevated MMP‐9 expression, and altered growth factor levels are major factors hindering the healing of DFUs, suggesting that targeting these factors could improve management and healing outcomes in DFU patients [113]. Growth factors such as VEGF and TGF‐β are pivotal in the healing process. In diabetic ulcers, the expression of these growth factors often becomes dysregulated. Zhang et al. [117] highlighted that recombinant human granulocyte/macrophage CSF treatment can enhance the production of growth factors necessary for tissue repair, underscoring the importance of growth factor signaling in managing diabetic ulcers [117].

MMPs are essential for appropriate epithelization and cell proliferation. Local mediators cause immune cells, fibroblasts, keratinocytes, and other wounded cells to release MMPs as part of normal wound healing. However, their dysregulation impairs epithelialization and is closely related to wounds that are difficult to cure. In diabetic ulcers, MMP activity is often dysregulated, leading to excessive matrix degradation and a failure to transition from the inflammatory phase to the proliferative phase. Recent studies revealed that higher levels of MMP‐1, MMP‐2, and MMP‐9 were associated with delayed wound healing in DFUs. Conversely, increased expression of MMP‐8 in tissues was linked to improved wound healing outcomes. Additionally, in healing DFUs, concentrations of MMPs (MMP‐1, MMP‐2, MMP‐8, MMP‐9) decreased, while levels of tissue inhibitors of metalloproteinases (TIMPs) such as TIMP‐1 increased, suggesting a potential balance between MMPs and TIMPs is crucial for effective wound healing [118]. These findings highlight the complex role of MMPs in DFU healing and suggest that modulating MMP levels or activity could offer therapeutic strategies for managing DFUs.

Dysregulated chemokine expression in diabetic individuals has been implicated as a contributing factor to delayed wound healing. RNA sequencing analyses have revealed a significant upregulation of several chemokines in diabetic skin compared with healthy skin, including CCL2, CCL7, CCL9, CCL12, CCL20, CXCL2, and CXCL15 [119]. Among these, CCL2 is notably elevated in diabetic wounds and plays a key role in immune cell chemotaxis throughout all phases of wound healing [119]. Conversely, diabetic wounds exhibit a marked deficiency in CXCL12, a chemokine essential for the recruitment of progenitor and stem cells during the proliferative phase. Studies have shown that exogenous administration of CXCL12 significantly enhances wound healing in diabetic mice [120], highlighting the critical role of stem cell homing and proper chemokine regulation in the restoration of skin integrity.

In addition, a study by Li et al. [121] explored the impact of chronic DLUs on macrophage phenotypes using scRNA‐seq. They found that persistent inflammation in DLUs is associated with an accumulation of M1 macrophages, which impair the transition to the M2 phenotype critical for resolution and healing. Insights into the differential expression patterns of genes associated with inflammation and healing were crucial in elucidating the challenges posed by chronic wounds in diabetic patients. Their findings advocate for novel therapeutic strategies that could enhance macrophage function and improve healing rates [121]. Furthermore, the work of Ma et al. [122] utilized scRNA‐seq to analyze macrophages in DLUs specifically. They identified distinct clusters of macrophages exhibiting different functional states and responses to the wound environment. Interestingly, the data indicated that a subpopulation of macrophages was engaged in promoting angiogenesis alongside inflammation, suggesting a dual role that could be pivotal in chronic wound management and healing. Immune dysregulation in DLUs involves a complex interplay among macrophages, T‐cells, miRNAs, cytokines, chemokines, growth factors, and MMPs. Understanding these interactions is essential for developing targeted therapies aimed at reversing the chronic inflammatory state, promoting proper immune function, and facilitating effective wound healing. Future research focusing on these pathways may yield innovative strategies to improve the management of DLUs, ultimately enhancing patient outcomes and quality of life.

### 4.3 Immune Dysregulation in Venous Leg Ulcers

VLUs arise from chronic venous insufficiency, leading to impaired blood circulation, increased venous pressure, and fluid buildup in the tissues, causing the skin to break down and subsequent ulceration in the lower extremities, particularly around ankles. The presence of immune dysregulation is a critical factor in the pathogenesis of VLUs, significantly affecting the healing process. Recent investigations highlighted roles of T‐cells, miRNAs, cytokines, growth factors, and MMPs in this complex dysfunction. Researchers found that in patients with VLUs, there is an altered T‐cells population with a predominance of proinflammatory Th1 cells and reduced Treg cells [123]. This imbalance leads to sustained inflammation and inhibits effective wound healing. In particular, low Treg cell levels correlate with prolonged healing times, as Treg cells are crucial for moderating immune responses and promoting resolution of inflammation. By targeting T‐cells populations or enhancing Treg cell activity, therapeutic strategies could be developed to facilitate healing in VLUs.

In the context of VLUs, cytokines play pivotal roles in both promoting and inhibiting the healing process and inflammatory cytokines such as TNF‐α, IL‐1β, and IL‐8, are often elevated. These cytokines maintain the inflammatory cascade, resulting in tissue damage and impaired healing. Yadav et al. [124] noted that levels of IL‐6 correlate with the size and duration of the ulcer, highlighting its role as a potential biomarker for ulcer prognosis. Additionally, chemokines like CCL2 (MCP‐1) promote the recruitment of monocytes and macrophages to the ulcer site, exacerbating inflammation if not regulated properly. Moreover, elevated levels of IL‐8 have also been observed in VLUs, contributing to neutrophil accumulation and tissue degradation [125]. Furthermore, the presence of IL‐1β has also been associated with increased MMP activity, which further degrades the ECM and inhibits wound closure [125]. This persistent inflammation can compromise the integrity of the ECM, impeding the healing process. On the other hand, the production of anti‐inflammatory cytokines, such as IL‐10, is often observed to be insufficient in VLUs. This imbalance between proinflammatory and anti‐inflammatory cytokines contributes to the persistence of inflammation and the failure to transition to the reparative phase of wound healing. According to Serag et al. [126], this dysregulation in cytokine signaling pathways not only impedes healing but also contributes to the recurrence of ulcers, highlighting the need for therapeutic strategies that aim to restore the cytokine balance in chronic wounds. Understanding the roles of these inflammatory mediators can guide the development of anti‐inflammatory treatments targeting their pathways.

In VLUs, the expression and activity of several key growth factors, such as VEGF and TGF‐β, are often dysregulated. Sánchez et al. [127] explored the role of recombinant human EGF (rhEGF) in managing VLUs, demonstrating that its application leads to enhanced granulation and promotes healing by stimulating cell proliferation and migration [127]. The findings suggest that growth factors like rhEGF can be valuable adjuncts in the treatment of VLUs, supporting a more efficient healing process. Furthermore, Barzegar et al. [123] indicated that an insufficient supply of growth factors due to local ischemia in chronic venous insufficiency can result in reduced angiogenesis, ultimately impairing the healing of VLUs. When biochemical signaling through growth factors is disrupted, the normal processes of angiogenesis and ECM synthesis are compromised, leading to delayed wound healing. Recent research findings indicated that growth factor treatments such as PDGF, bFGF, and EGF, significantly enhanced complete wound healing compared with placebo [128]. Additionally, these treatments led to a 48.8% greater reduction in wound area. No significant differences were observed in the overall adverse event rates between growth factor treatments and placebo [128]. However, the evidence quality was deemed low, suggesting that while growth factors show promise in improving VLU healing, further research is necessary to confirm these findings. The interplay between cytokines and growth factors is particularly crucial in the context of VLUs. Cytokines can modulate the expression of growth factors among local cells involved in wound healing. For instance, proinflammatory cytokines can enhance the expression of VEGF, resulting in increased angiogenesis; however, if the inflammatory response remains unchecked, it can lead to excessive tissue damage and further complications. Studies reported that a balanced expression of cytokines and growth factors is necessary for effective wound healing in VLUs. Dysregulation of these factors not only impairs cellular functions but also alters the wound microenvironment, fostering conditions that are unfavorable for healing. The focus on restoring this balance through pharmacological or biological therapies may provide new avenues for enhancing healing in patients with VLUs.

Moreover, researchers investigated the miRNA expression in VLU patients [129]. They found that overexpression of miR‐221, miR‐222, miR‐92a, and miR‐301a‐3p was found to hinder angiogenesis, while overexpression of miR‐296, miR‐126, miR‐378, and miR‐210 facilitated angiogenesis. Overexpression of miR‐34a/c, miR‐301a‐3p, miR‐450‐5p, miR‐424‐5p, miR‐516‐5p, and miR‐7704 increased local inflammatory responses and inhibited keratinocyte proliferation, impairing healing. Conversely, overexpression of miR‐19a/b and miR‐20 downregulated keratinocytes’ inflammatory response, promoting healing. Downregulation of miR‐205, miR‐96‐5p, and miR‐218‐5p enhanced cellular proliferation and promoted wound healing. Additionally, downregulation of miR‐17‐92 was linked with impaired healing [129].

In studies involving VLUs, MMPs are frequently implicated in the imbalance between matrix degradation and synthesis. This imbalance can lead to persistent inflammation, impaired tissue regeneration, and failure of the wound to close. In a study, the researchers evaluated the expression of MMPs in the wound fluids of VLUs through the application of a protease‐modulating wound dressing to VLUs and split‐thickness skin graft donor site wounds [130]. The dressing was designed to reduce excessive protease activity, particularly MMPs, which can hinder wound healing in chronic wounds. The dressing was applied to the wounds over a 12‐week period, and wound fluid samples were collected to analyze biomarker levels, particularly MMP‐2 and MMP‐9. The results revealed that MMP‐2 levels decreased significantly within the first 14 days of treatment and remained stable thereafter, though still higher than in acute wounds. MMP‐9 levels, however, showed minimal changes throughout the study. These findings suggest that the protease‐modulating wound dressing effectively reduced MMP‐2 activity, potentially promoting better healing in chronic wounds like VLUs, by improving the wound environment and preventing excessive protease degradation of the ECM [130]. These findings emphasize the importance of monitoring specific biomarkers, such as MMPs, in optimizing wound healing therapies for chronic conditions.

## 5 Immunomodulatory Strategies for Chronic Wound Healing

Treatment of chronic wounds can be challenging and often requires a multidisciplinary approach. Depending on the underlying cause, treatment may include wound debridement, dressings, antibiotics, compression therapy, and surgical interventions. It is also important to address any underlying medical condition, such as diabetes or vascular disease, to promote healing and prevent further complications. Controlling the immune response presents an appealing opportunity for developing new therapies as chronic wounds broadly result from excessive inflammation. To do this, a variety of material‐based and molecular techniques have been investigated, such as cytokines, protease inhibitors, miRNA, small interfering RNA (siRNA), and EVs that target the immune response.

### 5.1 Biomaterials

**FIGURE 1:**
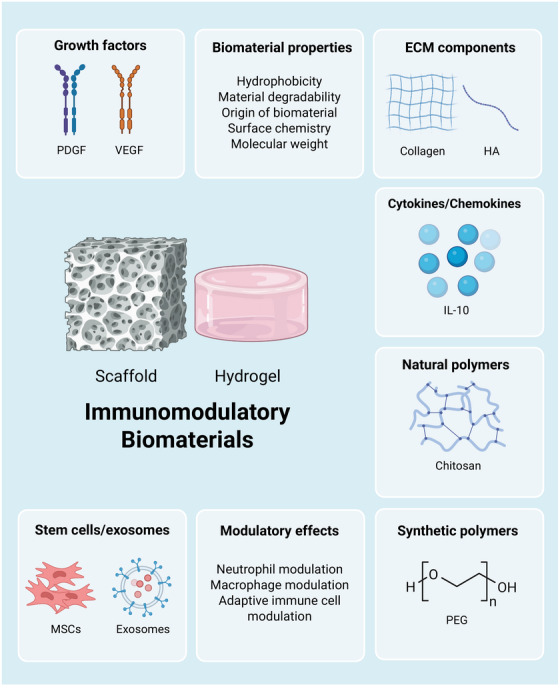
Schematic representation showing the incorporation of various bioactive components into biomaterials and hydrogels to modulate the immune microenvironment in chronic wounds. These components may include anti‐inflammatory cytokines, growth factors, immunoregulatory cells (e.g., MSCs), exosomes, peptides, and small molecules. The engineered systems aim to reduce chronic inflammation, promote macrophage polarization toward a prohealing, M2 phenotype, and support tissue regeneration and re‐epithelialization (created in BioRender).

Immunomodulatory biomaterials are specifically designed to modulate immune responses and foster a proregenerative microenvironment, making them particularly valuable in wound healing applications. A critical requirement for such materials is their ability to avoid triggering or sustaining a proinflammatory milieu. Instead, they should actively promote the polarization of macrophages toward the M2 reparative phenotype, which is essential for tissue regeneration. Therefore, the incorporation of immunomodulatory features should be considered a fundamental design criterion in the development of advanced wound healing therapies. Certain biomaterials are crafted to foster an anti‐inflammatory environment by engaging with certain pathways in order to promote the release of anti‐inflammatory cytokines, such as IL‐4, IL‐10, and IL‐13, and to facilitate the transition to tissue repair. Additionally, the biomaterial modulated the wound‐healing process by regulating TGF‐β1 and Smad7 mRNA expression, highlighting its anti‐inflammatory and immunoregulatory properties. However, excessive suppression of proinflammatory responses by biomaterials can disrupt the natural healing cascade, impairing macrophage activation, fibroblast function, and angiogenesis. This may lead to inadequate matrix synthesis, poor vascularization, and compromised tissue integrity. Achieving a balanced immune response remains a challenge, prompting research into strategies such as integrating anti‐inflammatory agents, modifying surface properties, and optimizing release kinetics to support effective wound healing. To enhance the immunomodulatory properties of biomaterials and hydrogels for chronic skin wound treatment, various bioactive components can be incorporated (Figure 1). These components can be ECM components (hyaluronic acid [HA], collagen, alginate), natural polymers (chitosan), synthetic polymers (polyethylene glycol‐PEG), growth factors (VEGF, PDGF), cytokines (IL‐10), and stem cells and their secretions (MSCs, exosomes).

Moreover, different biomaterials have been developed which specifically target different immune cells such as neutrophils, macrophages and adaptive immune cells. We have decided to group the biomaterials discussed in this work into the following categories to obtain immunomodulatory effects: (i) neutrophil modulation, (ii) macrophage modulation, and (iii) adaptive immune cell modulation.

#### 5.1.1 Neutrophil Modulation

Neutrophils are critical in the early immune response to biomaterials in wound healing, influencing their integration and overall healing outcomes. As the first responders, neutrophils secrete signaling molecules and form NET, which help modulate the immune environment. Their response is highly dependent on biomaterial properties and the surrounding microenvironment, shaping subsequent immune reactions. While neutrophils can facilitate tissue repair, excessive NET formation and inflammatory signaling may disrupt the healing process, highlighting the need for biomaterial designs that regulate neutrophil activity for optimal wound healing [131]. Mei et al. [132] formulated a silk fibroin hydrogel coencapsulating metformin‐loaded mesoporous silica microspheres and silver nanoparticles. In diabetic mouse models, this composite system inhibited NET formation, reduced the release of proinflammatory neutrophil mediators, and enhanced fibroblast migration and angiogenesis, collectively supporting more efficient wound healing [132]. Neutrophils recruitment and activation depend on the protein concentration on the biomaterial's surface, which is directly linked to its biocompatibility and surface properties. Nonporous biomaterials with low surface area‐to‐volume ratios often prolong inflammation, whereas nanofibrous networks, nanoparticles, and microporous structures enhance NET‐macrophage interactions, angiogenesis, and tissue regeneration [133].

Naturally derived polymers, resembling the ECM, promote functional immune responses that support regeneration without fibrosis [134]. For example, chitosan scaffolds enhance neutrophils presence and accelerate chronic wound healing [135], while fibronectin‐based hydrogels reduce neutrophil counts and expedite repair [136, 137]. Electrospun fibrous biomaterials, such as polydioxanone, minimize NET formation, particularly through TAK1 inhibition [138]. Additionally, incorporating silver [139], gold nanoparticles [140], or carbon nanotubes [141, 142] into naturally derived hydrogels effectively modulates neutrophils attachment and accelerates wound healing. Modifying biomaterial properties is a key strategy for regulating neutrophil adhesion and activation upon implantation. Hydrophilic, nonionic surfaces, such as those incorporating poly(ethylene glycol) (PEG), HA, glycosaminoglycans, chitosan, or heparin, reduce protein adsorption and proinflammatory cytokine production, thereby limiting leukocyte adhesion and macrophage fusion while promoting regenerative inflammation [143, 144, 145, 146, 147].

Similarly, synthetic polymers like poly(lactic acid), poly(lactic‐co‐glycolic acid) (PLGA), and poly(vinyl alcohol) enhance biocompatibility by minimizing protein adsorption and neutrophil recruitment [148, 149, 150]. Researchers demonstrated that encapsulating aspirin‐triggered resolvin D1 (AT‐RvD1) within a PLGA scaffold reduced neutrophil presence and migration in a murine dorsal skinfold model. This treatment also increased neutrophils, which contribute to vascular remodeling, while lowering the neutrophil‐to‐monocyte/macrophage ratio, indicating reduced inflammation and enhanced wound healing [151]. Another crucial aspect is material degradability which impacts biomaterial integration and tissue regeneration by influencing surface topography, charge, and protein adsorption, all of which affect neutrophil activation. Rapid or uncontrolled degradation can lead to toxic by‐products, triggering inflammation and excessive neutrophil recruitment [152]. To minimize immune responses and support regeneration, biomaterials should have a controlled degradation rate with bioresorbable by‐products. Natural polymers generally produce bioresorbable degradation products but degrade quickly, potentially provoking immune reactions. In contrast, synthetic polymers offer more stability but may lack biocompatibility. A hybrid approach combining natural and synthetic polymers allows fine‐tuning of degradation rates and mechanical properties. For example, grafting deferoxamine onto sodium alginate/bioglass hydrogel accelerates degradation, enhancing tissue penetration and promoting wound healing [153].

#### 5.1.2 Macrophage Modulation

Recent studies have explored therapeutic biomaterials to modulate the transition of macrophages from M1 to M2 to enhance cutaneous wound healing. For instance, researchers demonstrated that M2 macrophage polarization significantly improves wound closure and collagen deposition [154]. The contemporary biomaterial‐based approaches either target macrophage adhesion and recruitment or target their polarization to promote inflammation resolution. Natural biomaterials with immunomodulatory properties have underlined their regulatory influence on macrophage fate determination. A decellularized dermal scaffold (DDS), a skin‐derived tissue acting as framework for cellular ingrowth, has been proven to be a therapeutic for wound healing [155]. DDS can augment macrophage polarization through influencing their immunomodulation from the proinflammatory to anti‐inflammatory phenotype [155, 156]. Nanoparticles have also been investigated for macrophage modulation. For example, Konjac glucomannan‐modified SiO_2_ nanoparticles accelerated wound healing in both healthy and diabetic mice by inducing M1‐to‐M2 polarization, likely through mannose receptor clustering [157]. Similarly, silver nanoparticle‐loaded collagen‐chitosan scaffolds not only exhibited antimicrobial properties but also reduced inflammatory cell infiltration and CD68 expression [158]. Another approach involved encapsulating a miR‐223 5p mimic in HA‐based nanoparticles within a gelatin methacrylate (GelMA) hydrogel, which enhanced wound closure, increased collagen deposition, and promoted macrophage polarization toward an M2‐like phenotype [159].

Modifying the biomaterial's surface chemistry is one controlling approach for macrophage polarization. In fact, hydrophobic surfaces promote macrophage adherence. Although fewer macrophages and foreign body giant cells cling to hydrophilic/neutral surfaces but these cells release more cytokines than those that adhere to hydrophobic and ionic surfaces [160]. This might be the outcome of a particular activation of macrophages that are attached to the biomaterial and a gradual phenotypic switch. Furthermore, it has been shown that the chemical makeup of the biomaterial surface that monocyte‐derived macrophages are in touch which causes them to alter the expression of their surface proteins [161]. These results demonstrate the impact of biomaterial surface chemistry on macrophage responsiveness and activation, as numerous studies have supported these findings [162]. For instance, to promote cell adhesion, proliferation, and migration, surface topography has been changed to resemble the natural ECM structure by imprinting minute patterns on the surface. These patterns not only influence the activity of fibroblasts, ECs, and epithelial cells, but they also encourage a particular cell shape, which in turn promotes the polarization of anti‐inflammatory macrophages. For instance, smooth titanium surfaces promote the expression of inflammatory markers, while hydrophilic rough surfaces encourage an anti‐inflammatory state in macrophages [163]. Immunomodulatory characteristics are also influenced by the origin of the biomaterial. Synthetic biomaterials can be helpful as they typically prevent an unfavorable host immune response triggered by the antigens in naturally developed materials. For instance, the use of poly (methacrylic acid‐co‐methyl methacrylate) beads to treat diabetic mouse wounds expedited the healing of chronic wounds, most likely by boosting sonic hedgehog signaling (SHH) [164]. SHH has been linked to CD4+ T‐cells activation as well as increased keratinocyte and hematopoietic stem cells proliferation [165]. The ECM's natural components, such as HA, or its equivalents, such as chitosan, modulate the macrophage transition from the M1 to the M2 phenotype [166].

Biomaterials made from natural sources can replace the original ECM's missing parts more successfully. Hydrogel‐based materials have emerged as dynamic wound dressings that respond to the wound microenvironment and actively interact with skin tissues to support and accelerate healing. HA, chitosan, fibrin, keratin, alginate, and collagen are examples of naturally derived biomaterials that are frequently employed for healing of chronic wounds [167, 168, 169, 170, 171]. For instance, delivering neurotensin to diabetic wounds in rats using a chitosan‐based biomaterial stimulated rapid healing (50% wound area reduction) by lowering inflammatory cell numbers, levels of TNF‐α, and MMP‐9 at the site of damage [172]. Moreover, cellular behavior is significantly influenced by the physical and chemical characteristics of biomaterials. For example, a low molecular weight of HA activated M1 macrophages whereas a high molecular weight of HA led macrophages to exhibit anti‐inflammatory characteristics [173]. Collagen and highly sulfated HA‐based biomaterials facilitated transition of macrophages from the M1 to M2 phenotype and helped reduce inflammation. Xue et al. [174] synthesized sulfated HA (HA‐SO_3_) derivatives with a degree of substitution of 11.6% using HA with a molecular weight of 1 × 10⁵ g/mol. These derivatives significantly enhanced EC growth and migration, facilitated the phenotypic switch of vascular smooth muscle cells from a synthetic to a contractile state, and reduced macrophage adhesion and aggregation. Notably, HA‐SO_3_ also promoted macrophage polarization from the M1 phenotype to the anti‐inflammatory M2 phenotype, while exhibiting minimal hemolytic activity and reducing platelet aggregation [174]. Sulfated HA modulates the M1 and M2 repolarization via suppression of the NFκB pathway [175].

Building on this concept, Zhong et al. [176] developed a composite hydrogel by integrating sulfated HA with methacrylated gelatin and modified graphene (sHA/gelatin/G). When applied to a rabbit ear wound model, this hydrogel effectively promoted scarless wound healing through the suppression of fibrosis. Mechanistically, it increased the expression of anti‐inflammatory markers such as CD206 and IL‐10, while downregulating proinflammatory cytokines TNF‐α, IL‐1, and the fibrotic mediator TGF‐β, highlighting its immunomodulatory and antiscarring potential [176]. Duan et al. [177] developed adhesive hydrogels by combining DOPA‐modified HA with platelet‐rich plasma (PRP). The anti‐inflammatory effect comes from its later‐stage immunomodulatory and wound‐supporting properties, mainly through sustained PRP release as PRP is rich in growth factors (e.g., PDGF, TGF‐β, VEGF), which promote tissue repair and regulate immune responses, helping transition macrophages from an M1 to M2 phenotype in vivo. Second, through the HA component which is known to modulate inflammation by interacting with immune cells, maintaining hydration, and forming a protective barrier over the wound, thereby reducing infiltration of inflammatory stimuli [177]. Moreover, Hyalomatrix—a biodegradable dermal matrix composed of an esterified HA contact layer known as Hyaff (Medline)—stimulates cellular activity and ECM assembly in deep wounds. Among the most commonly used components in these formulations are alginate and collagen, both of which serve as effective bioactive agents in dermatological care. Alginate‐derived hydrogels are particularly valued for their high absorbency and hemostatic properties, making them ideal for managing exudative wounds. In contrast, collagen‐based hydrogels play a crucial role in stimulating collagen synthesis, a key element in tissue regeneration and the overall wound healing process. Integra, a 3D dermal matrix based of collagen and glycosaminoglycans, has demonstrated significant potential in accelerating wound healing in clinical settings [178]. Particularly effective for severe injuries—including those sustained by military personnel—Integra boasts a success rate of 78–86%. Its standout feature is its ability to facilitate robust dermal reconstruction, contributing to enhanced durability and mechanical strength, which plays a key role in managing complex wounds and supporting patient recovery [171]. Together, Integra and Hyalomatrix represent a synergistic and advanced solution in tissue engineering, offering a comprehensive strategy for treating challenging wound environments.

Growing evidence highlights that keratin has garnered significant attention in the field of wound dressings, owing to its exceptional properties—including hemostatic activity, anti‐inflammatory properties, and the ability to promote cell growth. Additionally, human hair keratin‐derived hydrogels‐based biomaterials have been employed in investigations on wound healing and have confirmed to be able to regulate inflammation [179]. Keratin‐based biomaterials have demonstrated the ability to promote M2 macrophage polarization in vitro, particularly in monocyte‐derived cell line models [179]. In a study by Shen et al. [170], keratin hydrogels were prepared by reconstituting lyophilized keratin extracts, derived from human hair fibers. The process involved the removal of hair fibers, followed by neutralization, centrifugation, filtration, purification, condensation, and finally freeze‐drying. Upon implantation, the hydrogels were found to have increased expression of mannose receptor (MMR) in M2 macrophages and they did not worsen the inflammation in vivo. Notably, MMR expression revealed a predominance of M2 macrophages, indicating an anti‐inflammatory immune environment [170]. These findings support the potential of keratin‐based biomaterials to therapeutically modulate macrophage polarization from a M1 phenotype to a regenerative M2 phenotype, offering promising strategies for enhanced tissue repair and wound healing.

Growth factors play a pivotal role in regulating wound healing and are frequently incorporated into hydrogel‐based wound dressings due to their potent biological effects. Among the most widely used growth factors are VEGF, PDGF, FGFs, and EGF [180]. Hydrogels loaded with growth factors effectively regulate macrophage polarization and enhance wound healing. For instance, VEGF encapsulated within a hybrid polyethylene glycol‐chitosan hydrogel increased the M2:M1 macrophage ratio, promoting hemostasis, wound closure, angiogenesis, and collagen deposition [180]. Other bioactive molecules, such as JK1 (a pH‐controlled H_2_S donor) [181] and prostaglandin E‐2 (PGE‐2) [182], have also demonstrated potential in shifting macrophages toward a prohealing phenotype.

Furthermore, stem cells are increasingly being integrated into biomaterial matrices to regulate macrophage polarization. Adipose‐derived MSCs (ADSCs) seeded on polycaprolactone electrospun fibers with mesh‐like structures enhanced IL‐10 expression, improving wound closure and collagen organization [183]. Similarly, bone marrow‐derived MSCs (BMSCs) embedded in hybrid hydrogels (polyester amide‐chitosan) accelerated re‐epithelialization, vascularization, and M2 macrophage polarization while reducing TNF‐α‐expressing M1 macrophages. Stem cell sheets, such as curcumin‐induced BMSC sheets, have also been shown to reduce M1 macrophages while upregulating M2‐associated markers like Relma and Arginase 1 [184]. Moreover, researchers explored the potential of electromagnetic biomaterials in addressing the challenges associated with immunomodulation of diabetic tissue repair [185]. Electromagnetic biomaterials are reported to facilitate the detection of diabetic wounds through their electric and magnetic properties, enabling real‐time monitoring and assessment of wound status. These biomaterials can influence macrophage polarization, encouraging a shift from the M1 phenotype to the M2 phenotype. The modulation of macrophage activity by these biomaterials contributes to a more favorable microenvironment for wound healing, addressing the chronic inflammation commonly observed in diabetic patients. These biomaterials contribute to enhancing the pathological microenvironment of diabetic wounds by reducing oxidative stress, modulating immune responses, and exhibiting antibacterial effects, which are crucial for effective wound healing. By regulating cellular behavior and supporting vascular and neural repair, electromagnetic biomaterials inherently promote tissue regeneration, addressing the impaired healing processes often observed in diabetic patients. Compared with conventional biomaterials, electromagnetic variants offer benefits such as noninvasiveness, deep tissue penetration, intelligent responsiveness, and the ability to synergize multiple stimuli, making them particularly suited for overcoming the complexities of diabetic tissue repair [185].

In the development of immunomodulatory biomaterials for wound healing, the incorporation of bioactive molecules plays a pivotal role in shaping the immune response and enhancing tissue repair. A wide array of bioactive agents—including anti‐inflammatory cytokines and growth factors such as VEGF and FGF—have been explored for their therapeutic potential. Uehara et al. [186] designed a gelatin methacryloyl hydrogel loaded with IL‐6, which was applied at the interface between the wound and a skin allograft. The controlled release of IL‐6 effectively reduced local inflammation and improved graft integration. Similarly, Chen et al. [187] developed electrospun poly(lactic acid) fibers capable of a cascade release of IL‐10. The early release mitigated the initial inflammatory surge, while sustained IL‐10 availability promoted macrophage polarization toward the M2 phenotype. Friedrich et al. [188] utilized a topical anti‐TNF‐α antibody in combination with HA in a rat burn model, observing reduced macrophage infiltration and IL‐1β expression shortly after injury. Das et al. [189] engineered an alginate‐based hydrogel delivering syndecan‐4 proteoliposomes and FGF‐2, which modulated macrophage phenotypes and cytokine profiles in favor of a regenerative response. Wang et al. [190] fabricated a composite hydrogel of HA, dextran, and β‐cyclodextrin loaded with resveratrol and VEGF plasmid, demonstrating suppressed IL‐1β and TNF‐α expression at the wound site. Last, Yang et al. [191] developed a HA‐based hydrogel infused with MSC‐derived EVs, which successfully induced M2 macrophage polarization and improved wound healing in a murine skin injury model. Collectively, these studies underscore the immense potential of integrating immunomodulatory molecules into biomaterials for effective chronic wound management. Moreover, researchers used immune cells for immunomodulation of chronic wounds. For example, Theocharidis et al. [192] loaded alginate dressings with polarized murine macrophages—or with the macrophage‐conditioned media (CM)—not only closed diabetic mouse wounds faster but also reshaped the local immune landscape. Immunohistology showed that cell‐laden bandages markedly increased total F4/80⁺ macrophage infiltration, with the greatest rise seen after M2c delivery. Within this larger pool of immune cells, M2 identifiers (YM1, CD206) were significantly enriched in M2a‐ and M2c‐treated wounds, whereas M1 markers (iNOS, TNF‐α) were unchanged or reduced. When concentrated CM was applied, the benefits were comparable: wounds contained fewer M1 macrophages yet maintained normal M2 numbers, indicating that soluble factors alone were sufficient to tilt macrophage polarization toward a reparative profile. Neutrophil counts (NIMP‐R14⁺) and T‐cells (CD3⁺) infiltration were not adversely affected, suggesting the intervention selectively modulated macrophage dynamics without broad immunosuppression. Collectively, these data highlight that both transplanted macrophages and their secretome restore a favorable M1/M2 balance, amplifying prohealing immune signals and thereby accelerating repair in diabetic wounds [192].

#### 5.1.3 Adaptive Immune Cell Modulation

The adaptive immune system, primarily driven by T and B lymphocytes, plays a crucial role in wound healing by providing a targeted and sustained response that resolves inflammation, promotes tissue repair, and prevents infection. While the innate immune response acts immediately upon injury, adaptive immunity fine‐tunes the healing process. CD4+ helper T (Th)‐cells and CD8+ cytotoxic T‐cells regulate immune cell activity through cytokine release, influencing macrophages, fibroblasts, and ECs [193]. Th1 cells enhance inflammation via IFN‐γ, whereas Th2 cells promote repair by secreting IL‐4 and IL‐13 to stimulate fibroblast activity and collagen synthesis [194]. Treg cells further support healing by secreting IL‐10 and TGF‐β to suppress excessive inflammation [78]. B cells, beyond their role in antibody production, contribute through cytokine secretion and immune regulation, aiding both inflammation resolution and tissue repair [195]. However, in chronic wounds, T‐cells exhibit a dysfunctional and unresponsive phenotype, characterized by an impaired ability to secrete key signaling molecules that are typically produced under normal physiological conditions [196].

Integrating adaptive immune mechanisms into biomaterial design presents a promising strategy for improving wound healing, particularly in chronic wounds where conventional therapies often fail. By harnessing the specificity and longevity of adaptive immunity, biomaterials can be engineered to modulate immune responses and enhance tissue repair. One key approach involves the controlled delivery of cytokines through biomaterials, ensuring sustained modulation of T and B cell responses. Cytokines such as IL‐10, TGF‐β, and IL‐4 promote a prohealing immune environment by reducing inflammation and supporting tissue regeneration. Research using chitosan‐based biomaterials for chronic wound healing have produced comparable outcomes. Yu et al. [197] developed a hydrogel composed of chitosan and poly [2‐(methacryloyloxy) ethyl] trimethyl ammonium chloride, which facilitated a shift from proinflammatory Th17 cells toward Treg cells. This shift played a crucial role in macrophage polarization toward an anti‐inflammatory phenotype, reducing excessive inflammation and fostering wound healing. In another study, a biodegradable cytogel incorporating IL‐33 within a physically cross‐linked DNA hydrogel enabled the sustained local release of IL‐33, promoting the recruitment of group 2 innate lymphoid cells and Treg cells. This mechanism proved effective in mitigating chronic inflammation and accelerating healing in diabetic wounds [198]. Additionally, Gong et al. [199] designed a CaH_2_ pulvis dressing capable of suppressing proinflammatory cytokine secretion while enhancing the infiltration of Treg cells, further supporting immune‐mediated wound healing. These studies highlight the potential of biomaterials tailored to regulate adaptive immune responses, offering new therapeutic avenues for treating complex wounds. Incorporating checkpoint inhibitors and costimulatory molecules into biomaterials offers a targeted approach to modulating T‐cells activity, particularly in wounds where excessive immune responses impede healing. By fine‐tuning T‐cells function, these biomaterials help suppress inflammation and create a more favorable environment for tissue repair. One example is the integration of programmed death‐ligand 1 (PD‐L1) into biomaterials, which interacts with the PD‐1 receptor on T‐cells to dampen their activity, reducing inflammation and promoting wound healing [200]. Su et al. developed a thermo‐responsive PF‐127 hydrogel that polymerizes at body temperature and steadily releases exosomal PD‐L1, effectively suppressing T‐cells activation and mitigating immune‐driven tissue damage [201]. Another innovative approach involves a wound dressing incorporating a photo‐crosslinking strategy with microcapsules for the controlled release of a TGF‐β inhibitor. This strategy successfully reduced CD4+ T‐cells infiltration and minimized scarring in both murine and larger animal models [202]. These advancements highlight the potential of immunoregulatory biomaterials in promoting wound healing by modulating adaptive immune responses. Table 2 summarizes the interactions between various biomaterials/hydrogels and different cell types used in chronic wound healing, including natural and synthetic polymers. Understanding these interactions is crucial for developing more effective wound healing strategies.

**TABLE 2:** Interactions between various biomaterials/hydrogels and immune cells in wound healing.

Biomaterials/hydrogels	Cell type	Interaction
Chitosan	Neutrophils	Enhanced IL‐8 levels and neutrophil migration, along with the regulation of neutrophil functions and inflammation, modulated through chemical modification of chitosan, specifically by altering its surface charge and hydrophobicity [203, 204]
	Macrophages	Induce anti‐inflammatory macrophage polarization and stimulate proinflammatory dendritic cells in the target tissue. Besides, chitosan enhances the secretion of IL‐8, MIP‐1, MCP‐1, and RANTES in macrophages [205]
Hyaluronic acid (HA)	Neutrophils	Decreased neutrophil migration, accompanied by the induction of anti‐inflammatory responses [206]
	T‐cells	Activation of Toll‐like receptors (TLRs) and T‐cells [206]
	DCs	Reduced dendritic cell (DC) activity, leading to the initiation of anti‐inflammatory responses [206]
Polycaprolactone (PCL)	Neutrophils	Elevated levels of IL‐2, IL‐4, and IgG, accompanied by increased neutrophil activity [207]
	Macrophages	Reduced proinflammatory cytokines, including TNF‐α, IL‐1, and IL‐6, while promoting anti‐inflammatory responses such as TGF‐β and IL‐4 [207]
Alginate	Macrophages	Modulation of the inflammatory phase through an increased presence of macrophages [208]
PLGA (polylactic‐co‐glycolic acid)	DCs	Elevated secretion of IL‐4, along with both proinflammatory and anti‐inflammatory cytokines [209]
	T‐cells	Enhanced antigen‐presenting cell (APC) activity and increased activation of CD8+ T‐cells [209]
Gelatin loaded with TLR 7/8 agonist	Macrophages	Effective macrophage repolarization, significantly enhancing proinflammatory markers and anticancer activity in cancer models [210]
Elastin	Macrophages	Increased M2 macrophages [211]
Polytetrafluoroethylene (PTFE)	DCs	Increased dendritic cell (DC) activity and heightened intensity of inflammatory responses
Calcium hydride (CaH_2_) pulvis	Treg cells	Suppressed proinflammatory cytokine secretion while enhancing the infiltration of Treg cells [199]
Keratin	Macrophages	Promoted M2 macrophage polarization in vitro, particularly in monocyte‐derived cell line models [179]
Poly(lactic acid)	Macrophages	Capable of a cascade release of IL‐10. The early release mitigated the initial inflammatory surge, while sustained IL‐10 availability promoted macrophage polarization [187]
Poly [2‐(methacryloyloxy) ethyl] trimethyl ammonium chloride	T‐cells	Facilitated a shift from proinflammatory T‐helper 17 (Th17) cells toward regulatory T (Treg) cells [197]
Electromagnetic	Macrophages	Influenced macrophage polarization [185]
Polyethylene glycol (PEG)	Macrophages	M1 macrophage markers (iNOS, COX2, and TNFα) within 3D PEG‐hydrogel were upregulated compared with those of cells growing on 2D [212].
Polyvinyl alcohol (PVA)/polyacrylamide	Macrophages	Promoted a shift in macrophages from a M1 phenotype to a prohealing and M2 phenotype in an in vivo model [213]
GBTF (composite hydrogel)	Macrophages	Facilitated fast wound healing through M2 polarization by activating PI3K/Akt signaling pathway and through proangiogenesis [214]

Recent advancements in hydrogel technologies have led to the development of innovative formulations that enhance the healing process. Sprayable hydrogels, such as those based on GelMA functionalized with antimicrobial agents, offer a noninvasive, uniform application method for irregular wound surfaces, improving antimicrobial activity and accelerating wound healing. Smart hydrogels, responsive to specific stimuli such as pH, temperature, or ion concentration, provide real‐time feedback and dynamic management of the wound environment, promoting personalized treatment. Cryogels, formed at sub‐zero temperatures, offer high porosity and enhanced bioactivity, making them ideal for encapsulating bioactive molecules and supporting cellular proliferation [215].

### 5.2 Cytokine‐Based Therapies

Chemokines are a specialized subset of cytokines with chemotactic properties, playing a central role in orchestrating the recruitment of immune cells to sites of tissue injury at precisely regulated times [216]. These signaling molecules are produced by both innate and adaptive immune cells in response to tissue damage or infection. To date, approximately 50 distinct chemokines have been identified, classified into four main subfamilies based on the arrangement of conserved cysteine residues: C‐motif ligand (CL), CC‐motif ligand (CCL), CXC‐motif ligand (CXCL), and CX3C‐motif ligand (CX3CL). Among these, the CCL and CXCL chemokines represent the most extensively studied and widely expressed groups. The term “cytokines” is used generically for a group of polypeptide growth factors, such as PDGF and VEGF, as well as ILs, and chemokines. In the last several years, locally released peptide mediators, including cytokines and polypeptide growth factors, have been studied for regulation of cell and tissue function, as many of these mediators are important in wound healing. Some may have autocrine, paracrine, or endocrine activity. Cytokines are an appealing target for therapeutic research since they are unquestionably important during the process of wound healing as these signaling molecules play essential roles in cell communication and immune responses.

The dysregulation of chemokine signaling, particularly during the critical transition from the inflammatory to the proliferative phase of wound healing, has garnered significant attention in the development of therapeutic biomaterials. Many researchers have focused on designing biomaterials that favor M2 macrophage polarization to promote tissue repair and resolution of inflammation. However, while M2 macrophages are generally considered beneficial for wound healing, their excessive activation can also contribute to adverse outcomes such as macrophage fusion, fibrosis, and the foreign body response, potentially leading to excessive scarring [217]. Therefore, the immunomodulatory design of biomaterials must strike a balance to support regeneration while avoiding pathological remodeling. One approach to assess whether a biomaterial promotes M2 macrophage polarization is by evaluating its impact on chemokine signaling. However, our current understanding of chemokines in wound healing is largely based on animal models, which often differ from humans in chemokine expression. While chemokines like CCL2, CCL5, CXCL8, and CXCL12 have been relatively well‐studied, the roles of many others remain unclear, with inconsistent classification as pro‐ or anti‐inflammatory. Generally, chemokines such as CXCL4–11, CCL1–5, 7, 11, 12, 20, 24, 26, XCL1–2, and CX3CL1, are linked to proinflammatory responses, promoting recruitment of neutrophils, eosinophils, and leukocytes. In contrast, CXCL12, 13, 21 and CCL8, 13, 14, 17–19, and 27 are more often associated with anti‐inflammatory roles, supporting stem cell homing, angiogenesis, and ECM remodeling. Notably, CXCL1, 2, 5, and CCL22 exhibit both pro‐ and anti‐inflammatory functions, highlighting the complexity of chemokine signaling in wound healing [218, 219]. IL‐37 is reported to be a natural suppressor of innate inflammation and has shown promise in enhancing the healing process of diabetic wounds. In diabetic mouse model, IL‐37 administration accelerated wound closure by inhibiting the mitogen‐activated protein kinase (MAPK)/NLRP3 signaling pathways, leading to reduced expression of proinflammatory cytokines such as TNF‐α and IL‐1β [220]. This suggests that IL‐37 may offer a novel therapeutic approach for managing chronic wounds associated with diabetes.

**FIGURE 2:**
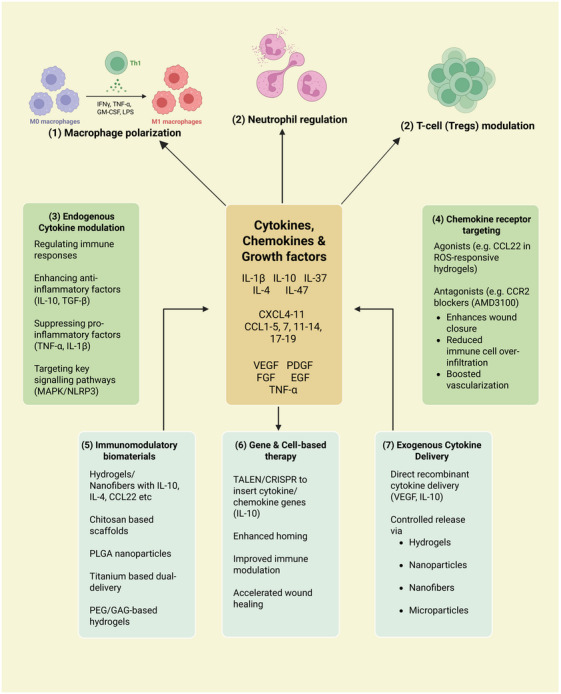
Overview of cytokine‐based immunomodulatory strategies for chronic wound healing. This schematic illustrates the multifaceted approaches utilizing cytokines to modulate immune responses in chronic wound environments. Therapeutic strategies include: (1) macrophage polarization, focusing on shifting macrophages from a M1 phenotype to a prohealing M2 phenotype using specific cytokines; (2) neutrophil and T‐cells regulation, involving targeted modulation of neutrophil lifespan, NET formation, and T‐cells subsets (e.g., regulatory T‐cells, Th17) to prevent chronic inflammation; (3) endogenous cytokine modulation, aiming to restore the balance of pro‐ and anti‐inflammatory cytokines naturally through microenvironmental conditioning; (4) chemokine receptor targeting in wound healing. Delivery of CCL22 (CCR4 agonist) via ROS‐responsive hydrogels promotes immune regulation. CCR2 antagonists (e.g., AMD3100) reduce immune cell overinfiltration, enhance vascularization, and accelerate wound closure; (5) immunomodulatory biomaterials loaded with cytokines, such as hydrogels and scaffolds that allow for localized and sustained release of therapeutic cytokines at the wound site; (6) gene and cell therapies, including viral or nonviral delivery of cytokine genes to improve immunomodulation and to enhance regeneration; (7) exogenous cytokine delivery, involving the direct administration of recombinant cytokines (e.g., GM‐CSF, IL‐1Ra) to modulate immune cell activity and promote tissue repair. Collectively, these strategies aim to reprogram the chronic wound microenvironment toward a proregenerative immune phenotype, supporting resolution of inflammation and restoration of skin integrity (created in BioRender).

Cytokine‐based immunomodulation can be classified as endogenous cytokine modulation or exogenous cytokine delivery. Endogenous cytokine modulation aims to rebalance the local immune environment by influencing the body's own cytokine production. In chronic wounds, this involves reducing elevated levels of proinflammatory cytokines such as TNF‐α and IL‐1β, while enhancing anti‐inflammatory cytokines like IL‐10 and TGF‐β. Strategies such as promoting M2 macrophage polarization, regulating T‐cells responses, or using small molecules to target key signaling pathways can help restore immune equilibrium and support healing. In contrast, exogenous cytokine delivery involves the direct administration of specific cytokines or growth factors to the wound site. Recombinant cytokines including IL‐10, GM‐CSF, and VEGF, have been utilized to reduce inflammation, stimulate angiogenesis, and promote tissue regeneration. Advanced biomaterials such as hydrogels and nanoparticles enable localized and sustained release of these cytokines, enhancing their therapeutic potential while minimizing systemic effects (Figure 2).

#### 5.2.1 Exogenous Cytokine Delivery

One of several chemokine‐based therapies involves the direct delivery of recombinant or synthetic chemokines to the wound site to promote cell recruitment and tissue regeneration. For instance, Qu et al. [221] designed a ROS‐responsive hydrogel patch that delivers CCL22 in response to elevated ROS levels typically found in diabetic wounds. This hydrogel not only scavenges excessive ROS but also recruits Treg cells to the wound site, promoting anti‐inflammatory responses and accelerating wound closure in diabetic mouse model. Conversely, chemokine receptor antagonists can be employed to mitigate excessive immune cell infiltration that exacerbates chronic inflammation. Antagonists targeting receptors such as CCR2 and CXCR4 have demonstrated efficacy in reducing monocyte and neutrophil accumulation, respectively, aiding in the resolution of persistent inflammatory responses. CXCR4 plays a pivotal role in recruiting various immune cells to wound sites. Studies have demonstrated that antagonizing CXCR4 can accelerate wound healing by mobilizing endothelial progenitor cells and enhancing fibroblast and macrophage activity. For example, a study involving AMD3100, a CXCR4 antagonist, showed significant improvements in wound closure, collagen formation, and vascularization in diabetic mice. The treatment also increased the presence of monocytes/macrophages at the wound site, contributing to enhanced healing processes [222]. Moreover, CXCL5, through its receptor CXCR2, influences inflammatory responses and neovascularization. Therefore, neutralizing CXCL5 has been shown to improve neovascularization and accelerate wound healing in diabetic models. A study reported that CXCL5 neutralizing antibodies upregulated VEGF and SDF‐1, promoting endothelial progenitor cell function and enhancing wound healing processes [223]. For macrophage polarization, cytokines with anti‐inflammatory potential such as IL‐10 were also investigated. Although initial results from clinical studies were not sufficient, recombinant IL‐10 has been added to a dextrin nanogel matrix to treat chronic wounds [224]. In addition, a synthetic lipopeptide, namely macrophage‐activating lipopeptide‐2 (MALP‐2) enhanced wound healing in diabetic mice by boosting macrophage infiltration and guiding M2 polarization. Also, favorable outcomes were seen in a phase I clinical trial employing MALP‐2 to treat diabetic wounds in 12 individuals [225]. Growth factors such as PDGF‐BB, VEGF‐A, and heparin‐binding EGF‐like growth factor (HB‐EGF) are particularly interesting as therapeutic targets because of their signaling potential and their capacity to engage with a variety of cell types in the wound environment. Michael and his colleagues showed that a therapy of PDGF‐BB, VEGF‐A, and HB‐EGF significantly increased re‐epithelialization and granulation tissue in a NOD mice model of type 1 diabetes (T1D). Also, they discovered that in the NOD mouse model, variations in the cellular milieu of a wound, such as varying concentrations of M1 macrophages, M2 macrophages, and effector T‐cells, are most predictive of wound‐healing success. Their findings imply that the triple growth factor therapy may be a useful treatment for chronic nonhealing wounds that develop as a result of diabetes [226].

Gene therapy approaches represent another avenue of intervention, utilizing plasmids or viral vectors to locally regulate chemokine expression. Advancements in gene editing have enabled the modification of stem cells to secrete beneficial factors at wound sites. Stem cell therapies further benefit from chemokine modulation. For instance, a study utilized transcription‐activator‐like effector nuclease to insert CXCR4 and IL‐10 genes into amniotic MSCs. The modified cells exhibited enhanced angiogenic and anti‐inflammatory properties, leading to accelerated wound healing in diabetic models [227]. Moreover, genetic modification of MSCs to overexpress CCR2 enhances their responsiveness to CCL2, a ligand that attracts monocytes and modulates macrophage polarization. Studies have demonstrated that CCR2‐engineered MSCs exhibit improved homing to injury sites, reduced monocyte infiltration, and a shift toward a reparative M2 macrophage phenotype, collectively promoting tissue repair in diabetic wound models [119].

Biomaterials have emerged as pivotal tools in modulating chemokine signaling to enhance immunomodulation and accelerate healing in chronic wounds. By integrating natural and synthetic biomaterials with bioactive agents, researchers aim to create environments that promote effective immune responses and tissue regeneration. Hydrogels, nanofibers, and scaffolds can be designed to release chemokines in a spatially and temporally controlled manner, facilitating targeted immune cell recruitment and phenotype switching. For example, chitosan or alginate hydrogels loaded with CXCL12 support stem cell migration and angiogenesis, while electrospun fibers delivering IL‐10 or CCL22 promote M2 macrophage polarization. Studies highlighted chitosan as a natural biomaterial with significant immunomodulatory properties. One of its primary immunomodulatory effects is its ability to influence neutrophil migration, which is critical in the early inflammatory phase of wound healing. Studies indicate that chitosan, depending on its degree of acetylation, can enhance or inhibit neutrophil migration. Lower acetylation levels of chitosan have been associated with increased neutrophil migration, making it a potential candidate for managing the inflammatory response in chronic wounds [228]. Furthermore, chitosan has been shown to modulate the secretion of CXCL8 (IL‐8), a chemokine crucial for neutrophil recruitment to the wound site. In chronic wounds, where neutrophil function is often impaired, this regulation of CXCL8 can facilitate the early‐stage immune response, promoting effective wound healing. Moreover, IL‐4 has emerged as a key player in tissue regeneration. It activates lymphocytes and promotes the polarization of macrophages toward the M2 phenotype, which is crucial for tissue repair. IL‐4 also stimulates the proliferation and differentiation of various cell types, contributing to efficient tissue regeneration. Recent studies have highlighted the potential of IL‐4‐loaded hydrogels in promoting immunomodulation and tissue repair. An injectable hydrogel scaffold enabling sustained IL‐4 release effectively enhanced M2 macrophage and Th2 immune responses [229]. Similarly, titanium‐based biomaterials combined with hydrogels have shown promise, where an initial burst of IFN‐γ followed by sustained IL‐4 release from nanotubes mimicked the natural immune sequence, guiding macrophage polarization toward the reparative M2 phenotype [229]. These findings underscore the synergistic potential of IL‐4 and hydrogel‐based systems in advancing chronic wound healing and regenerative therapies. PLGA electrospun fibers containing IL‐10 facilitated macrophage polarization from M1 to M2 phenotype [187].

Advanced delivery systems utilizing microparticles and nanoparticles have been developed to administer cytokines and growth factors effectively for immunomodulation. Nanoparticles, particularly those composed of biodegradable polymers like PLGA, have been employed to encapsulate and deliver cytokines in a controlled manner [230, 231]. Their small size, biocompatibility, and ability to encapsulate a variety of bioactive molecules make them ideal candidates for enhancing wound healing. Polymeric nanoparticles can be designed to encapsulate cytokines such as TGF‐β, IL‐10, IL‐4, and TNF‐α. The sustained and controlled release of these cytokines through nanoparticles helps in maintaining an optimal balance between proinflammatory and anti‐inflammatory responses, crucial for effective wound healing. Further, another potential chemokine to facilitate wound healing is SDF‐1. Delivering nanosized SDF‐1 liposomes to diabetic mouse wounds boosted dermal cell proliferation, synthesis of granulation tissue, and reduced wound closure duration [232]. Additionally, SDF‐1 is shielded by liposomes from being destroyed by proteases and serine exopeptidase. The efficacy of such SDF‐1 treatments still needs to be improved for example with a better delivery strategy for clinical application.

#### 5.2.2 Endogenous Cytokine Modulation

Chemokines can bind to ECM glycosaminoglycans like heparin and chondroitin sulfate through electrostatic interactions between their positively charged residues and the negatively charged sulfate groups [233]. Leveraging this property, several biomaterial‐based strategies have been developed to modulate inflammation in chronic wounds. Researchers designed an anti‐inflammatory hydrogel composed of heparin and star‐shaped PEG that effectively sequestered inflammatory chemokines—such as IL‐8, MCP‐1, MIP‐1α, and MIP‐1β—from diabetic wound fluid [234]. This hydrogel reduced monocyte and neutrophil migration, alleviated inflammation, and enhanced granulation tissue formation and vascularization within 10 days, even in highly inflamed wounds. In animal models, this hydrogel demonstrated superior performance compared with Promogran, a commercially available anti‐inflammatory dressing, by more effectively reducing inflammation, enhancing angiogenesis, and accelerating wound closure [234]. Similarly, investigators developed a Cu_5_._4_O/Hep/PEG hydrogel capable of capturing various inflammatory chemokines, leading to suppressed macrophage and neutrophil migration and decreased inflammatory signaling [235]. This finding highlight the potential of glycosaminoglycan‐based hydrogels in regulating chemokine activity for immunomodulation and improved healing in chronic wounds.

In addition, IL‐1β is an anti‐inflammatory cytokine that plays a crucial role in wound healing by modulating the immune response. For instance, IL‐1β participates in a proinflammatory positive feedback loop that maintains a prolonged proinflammatory wound macrophage phenotype and impairs diabetic wound healing. An antibody to block IL‐1β was shown to significantly expedite wound closure in diabetic mice in a study by Mirza et al. [236] by decreasing proinflammatory macrophages and increased prohealing factors. Another method of preventing IL‐1β signaling pathways is by using IL‐1 receptor antagonist (IL‐1Ra). Notably, the interaction between IL‐1β and IL‐1Ra, acting upon the IL‐1 receptor (IL‐1R), plays a pivotal role in promoting the healing of epithelial wounds. Moreover, it has been observed that the ratio of IL‐1Ra to IL‐1 in healing wound fluids is significantly higher compared with that in fluids from chronic wounds (480:1 versus 7:1) [237]. Unsurprisingly, it has been demonstrated that utilizing IL‐1Ra to suppress IL‐1 speeds up wound healing in diabetic mouse corneas [234]. Proinflammatory cytokines like IL‐1β and TNF‐α play significant roles in the inflammatory phase of wound healing. Targeted inhibition of these cytokines has been explored to control excessive inflammation and facilitate tissue repair. Etanercept, a recombinant fusion protein combining the extracellular domain of the human TNF receptor 2 (p75) with the Fc portion of IgG1, acts as a decoy receptor for TNF‐α, thereby neutralizing its activity. A study demonstrated that Etanercept reduced the cytotoxic effects of chronic wound fluid on fibroblasts by approximately 30% and neutralized TNF‐α binding by up to 80%. These findings suggest that direct application of Etanercept to chronic wounds may diminish TNF‐α‐mediated inflammation, potentially reducing wound chronicity and promoting healing [238]. Canakinumab, a human monoclonal antibody targeting IL‐1β, has been shown to effectively reduce neutrophilic inflammation. In a case study of a patient with Schnitzler syndrome, treatment with Canakinumab led to a rapid decrease in dermal neutrophil numbers and expression of proinflammatory cytokines IL‐1β, IL‐8, and IL‐17 within 1 month of initiation. Although this study focused on Schnitzler syndrome, the results indicate that Canakinumab's inhibition of IL‐1β can swiftly reduce neutrophilic infiltration and associated inflammation [239]. These findings suggest potential applications of Canakinumab in managing chronic wounds characterized by excessive neutrophilic activity. In parallel, monoclonal antibodies targeting proinflammatory chemokines, such as anti‐TNF‐α or anti‐CCL2, have shown therapeutic benefits by dampening the inflammatory response when used in conjunction with delivery vehicles like HA‐based hydrogels. HA hydrogel containing anti‐TNF‐ α decreased IL‐1β levels and also decreased macrophage infiltration [240]. Additionally, EV‐based therapies derived from MSCs have shown promise in modulating chemokine pathways. These MSC‐EVs, often delivered via hydrogel systems, can enhance M2 macrophage polarization, stimulate ECM remodeling, and promote angiogenesis, which is discussed in detail in the Section 5.5.

Sequential distribution of pro‐ and anti‐inflammatory cytokines may be a smart move to facilitate the healing of chronic wounds because the extended presence of proinflammatory cytokines may hinder resolution and both pro‐ and anti‐inflammatory cytokines are required for acute wound healing. This idea has been investigated in different tissues with some success. For instance, decellularized bone was created to sequentially release IFN‐γ and IL‐4 and implanted in mice at the damage site to enhance bone regeneration. Improved wound healing was the result of macrophage polarization, which changed their phenotype from pro‐ to anti‐inflammatory due to sequential release of IFN‐γ and IL‐4 [241]. Considering the pivotal role of the IFN‐γ and TGF‐β signaling pathway in modulating inflammatory responses during the process of wound healing, it is reasonable to propose that employing a similar approach could be viable for addressing the challenges posed by chronic wounds. Emerging innovations include the development of smart dressings capable of releasing chemokines in response to specific wound cues such as pH or enzymatic activity, and the potential application of CRISPR‐based tools for in situ regulation of chemokine gene expression. Future strategies may involve tailored chemokine cocktails designed to meet the dynamic needs of each wound healing phase, offering a more precise and effective therapeutic approach for chronic wounds.

### 5.3 Protease Inhibitor‐Based Therapies

Protease inhibitor‐based therapy is emerging as a critical strategy for managing chronic wounds, primarily due to the significant role proteases play in the pathological modulation of wound healing processes. Chronic wounds are often characterized by elevated levels of MMPs, cathepsin G and neutrophil elastase (NE), which contribute to delayed wound closure. Dysregulation of MMPs and their inhibitors (TIMPs) plays a key role in impaired wound healing [118]. In chronic wounds, elevated MMP levels—driven by increased neutrophil and macrophage infiltration—lead to excessive degradation of essential growth factors, cytokines, and ECM components, disrupting tissue repair. Protease inhibitors target these enzymes to rebalance the wound environment and stimulate healing. Several protease inhibitors have been investigated for their potential use in chronic wound healing, including, cysteine protease inhibitors, serine protease inhibitors, and metalloproteinase inhibitors. These different protease inhibitors are discussed in this review. One approach involves the use of TIMPs, endogenous proteins that regulate MMP activity. Modulating TIMP levels has shown promise in controlling elevated MMP concentrations, thereby influencing wound healing outcomes. Additionally, bioresponsive hydrogels that release MMP inhibitors in response to local enzyme levels have been developed, offering targeted modulation of protease activity in the wound environment [242]. Drupin, a cysteine protease, have shown potential for promoting excisional wound healing in mice as an herbal wound care. Researchers found that Drupin promoted wound healing when applied directly on wound as solutions, by downregulation of MMP‐9, enhanced expression of arginase‐1 in macrophages and accelerated collagen synthesis at wound site [243, 244]. Despite being a protease itself, Drupin has been reported to modulate wound healing by downregulating MMP‐9 (a MMP), which may indirectly act like a metalloproteinase regulator. In another study, ND‐336, which specifically inhibits MMP‐2, MMP‐9, and MMP‐14, was applied to speed up the healing of wounds in diabetic mice by reducing inflammation, promoting re‐epithelialization, and boosting angiogenesis [245].

Cysteine protease inhibitors are also a promising therapeutic strategy for managing chronic wounds, particularly by targeting cathepsins—lysosomal cysteine proteases implicated in excessive ECM degradation and sustained inflammation. In diabetic wound models, inhibition of cathepsin K using agents like odanacatib and cathepsin K inhibitor‐II has demonstrated accelerated wound closure [246]. Specifically, treatment with these inhibitors significantly reduced the wound area compared with controls, suggesting enhanced reepithelialization and tissue repair. Beyond cathepsin K, other cathepsins such as B, L, and S have been identified as contributors to chronic wound pathology. Their overexpression correlates with impaired healing, making them attractive targets for therapeutic intervention. Further insights into the role of cysteine proteases in wound healing were provided by a study examining the interaction between cathepsin D and iron ions under varying pH conditions. The research revealed that at physiological pH, specific residues in cathepsin D exhibited a strong affinity for ferrous ions, potentially inhibiting the enzyme's proteolytic activity. Conversely, under acidic conditions typical of inflamed wounds, different residues mediated the interaction, suggesting a pH‐dependent regulatory mechanism [247]. These findings highlight the complexity of protease regulation in the wound environment and suggest that modulating factors like pH and metal ion availability could influence protease activity and, consequently, wound healing outcomes. In addition to synthetic inhibitors, natural cysteine protease inhibitors were investigated. A study explored the effects of tick‐derived cysteine protease inhibitors, such as Sialostatin L and Sialostatin L2, in a mouse model of psoriasis‐like skin inflammation. These inhibitors significantly reduced clinical symptoms and histological markers of inflammation, including epidermal thickness and immune cell infiltration [248]. The treatment also modulated the expression of proinflammatory cytokines, suggesting that such natural inhibitors can effectively suppress excessive immune responses and may have therapeutic potential in inflammatory skin conditions. Advancements in targeted delivery systems have further enhanced the potential of cysteine protease inhibitors. Researchers developed antibody‐peptide conjugates that deliver covalent inhibitors specifically to cells expressing target proteases. This approach allows for selective inhibition of cysteine cathepsins in specific cell types, minimizing off‐target effects and enhancing therapeutic efficacy [249]. Such targeted strategies could be particularly beneficial in wound healing applications, where localized modulation of protease activity is desired. Moreover, the development of thermo‐responsive hydrogels incorporating cysteine‐based compounds has opened new avenues for wound care. For instance, a study introduced a hydrogel containing N‐acetylcysteine, which demonstrated enhanced wound healing properties in dermal and oral ulcer models. The hydrogel facilitated the reduction of inflammation which highlights the potential of cysteine‐containing biomaterials in wound management [250]. However, the development of selective inhibitors faces challenges due to the overlapping substrate specificities and essential physiological roles of these enzymes. To address specificity and minimize off‐target effects, innovative delivery systems are being investigated. For instance, nanoparticle‐based formulations have been developed to codeliver protease inhibitors alongside growth factors, protecting the latter from degradation in the protease‐rich environment of chronic wounds [251]. Such approaches have shown promise in preserving growth factor activity and enhancing wound healing outcomes in preclinical models. While these findings underscore the therapeutic potential of cysteine protease inhibitors in chronic wound management, further research is needed to optimize their specificity, delivery mechanisms, and clinical efficacy.

Protease inhibitors modulate immune responses in chronic wounds through several interconnected mechanisms that restore tissue homeostasis and promote healing. One key mechanism involves preserving the integrity of the ECM. In chronic wounds, excessive protease activity leads to ECM degradation, impairing the structural framework necessary for cell migration and tissue regeneration. By inhibiting proteases, these inhibitors prevent ECM breakdown, thereby maintaining a scaffold that supports immune cell infiltration and tissue repair processes. Recent studies have demonstrated the efficacy of protease inhibitors in preserving ECM integrity. For instance, recombinant TIMP‐2 has been shown to promote wound healing by suppressing MMPs and inflammatory cytokines in corneal epithelial cells. In a study by Folorunso et al. [252], recombinant TIMP‐2 treatment significantly promoted wound closure and reduced the expression of IL‐1β, IL‐6, IL‐8, and TNF‐α, as well as MMPs like MMP‐1, MMP‐2, MMP‐3, MMP‐9, MMP‐10, and MMP‐13 in corneal epithelial cells. This suggests that TIMP‐2 facilitates wound healing by attenuating inflammation and protease‐mediated tissue degradation, indicating its potential therapeutic application in wound management. Additionally, protease inhibitors protect crucial cytokines and growth factors from proteolytic degradation. This preservation ensures the availability of signaling molecules essential for the polarization of macrophages toward the M2 phenotype and for the proliferation of keratinocytes. One approach involves the use of heparan sulfate analogues, which are engineered polymers designed to mimic the properties of natural heparan sulfates. These analogues can sequester growth factors and cytokines within the ECM, protecting them from proteolytic degradation. By maintaining the local presence of these signaling proteins, heparan sulfate analogues support the preservation of anatomical form and function, contributing to tissue repair and regeneration. Importantly, these analogues are resistant to enzymatic degradation, enhancing their stability and efficacy in the wound environment [253].

Recent studies have elucidated the role of protease inhibitors in modulating neutrophil‐mediated inflammation, particularly through the inhibition of enzymes like NE. Excessive NE activity contributes to the formation of NETs, which, while integral to the immune response, can exacerbate inflammation and impede tissue repair when overproduced. By attenuating NE activity, protease inhibitors reduce NET formation, thereby mitigating prolonged inflammation and promoting more efficient wound healing. For instance, a study demonstrated that the serine protease inhibitor derived from *Trichinella spiralis* effectively inhibits NE activity, leading to impaired NET formation and reduced inflammatory responses in human neutrophils [254]. This highlights the potential of serine protease inhibitor derived from Trichinella spiralis (TsSERP) as a therapeutic agent in controlling excessive neutrophil activation and NET‐associated tissue damage [254]. Additionally, research has shown that endogenous proteases can induce a nonclassical form of NETs via activation of protease‐activated receptor 2 (PAR2). Preincubation with protease inhibitors was found to prevent this NET formation, suggesting that targeting specific protease pathways can modulate NET‐mediated inflammation [255]. Moreover, serine protease inhibitors (Serpins) such as α1‐antitrypsin (Serpin A1), antiplasmin (Serpin F2), and C1‐inhibitor (Serpin G1) play crucial roles in modulating immune responses during wound healing. In diabetic wounds, decreased levels of these Serpins correlate with increased NE activity and impaired healing. Restoring Serpin levels via EVs has been shown to accelerate wound closure in mice chronic wound models, suggesting their potential as therapeutic agents [256]. Additionally, low‐dose trypsin has been found to enhance wound healing by activating PAR2, leading to increased fibroblast and macrophage migration, adhesion, and proliferation. This activation upregulates genes associated with cell proliferation and ECM formation, contributing to tissue regeneration [255].

Recent studies have explored the impact of protease inhibitors on macrophage polarization, particularly the transition from the M1 phenotype to the prohealing M2 phenotype. For instance, bone morphogenetic protein 7 treatment has been shown to enhance M2 macrophage numbers and decrease the M1/M2 ratio in chronic wounds of streptozotocin‐induced diabetic mice, indicating reduced inflammation and improved healing outcomes [257]. Moreover, targeting Pim2, a kinase involved in metabolic reprogramming, alleviated inflammatory arthritis by inhibiting glycolysis and reducing M1 macrophage polarization [258]. This indicates that metabolic pathways can influence macrophage phenotypes, and their modulation may have therapeutic benefits. Similarly, CXCR4 silencing has been shown to inhibit glycolysis, enhance mitochondrial activity, and promote M2 macrophage polarization [259]. These findings collectively suggest that modulating protease‐related and metabolic pathways can influence macrophage transitions. Although further research is needed to directly connect protease inhibitors with macrophage polarization in chronic wounds, current evidence strongly supports their potential role in orchestrating immune resolution and tissue regeneration through this mechanism.

Proteases play a significant role in modulating DC and T‐cells functions, thereby influencing antigen presentation and T‐cells activation. Proteases, such as cathepsins, are involved in the processing of antigens within DCs, which is crucial for the presentation of peptides via MHC molecules to T‐cells. For instance, cathepsin S has been shown to be essential for the maturation of MHC class II molecules during antigen presentation, and its activity affects DC‐mediated T‐cells activation. Moreover, studies have demonstrated that PAR‐2 signaling, triggered by serine proteases, stimulates the development of DCs from bone marrow progenitor cells, indicating that protease activity can influence the differentiation and function of antigen‐presenting cells (APCs) [260]. By modulating protease activity, inhibitors can help steer the immune response toward a regenerative phenotype. For example, the use of serine protease inhibitors has been shown to affect DC development, suggesting that controlling protease activity can influence the maturation and function of these cells, which are pivotal in initiating T‐cells responses. Additionally, the regulation of protease activity can impact the activation of T‐cells by affecting antigen processing pathways within DCs, thereby enhancing the overall healing process through a more controlled and regenerative immune response [261].

**FIGURE 3:**
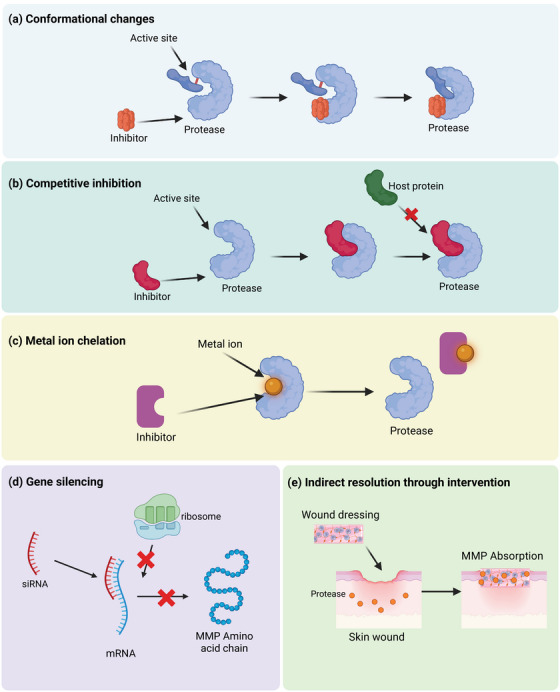
Strategies to modulate protease activity or expression. (a) Induction of conformational changes in a protease by binding of an inhibitor to any site blocking the active site for any activating protein. (b) Competitive inhibition of protease activity by binding of an inhibitor making it unavailable for an activating protein. (c) Chelating agents bind to metal ions within the protease's active site, thereby inhibiting its activity. (d) MMP genes can be silenced by siRNA molecules that cause the endonucleolytic cleavage of the target mRNA molecules that suppress translation of mRNA of proteases. (e) Any intervention that promotes wound healing can indirectly contribute to the regulation of protease activity (created in BioRender).

Recent advancements have introduced innovative strategies to modulate protease activity and expression. An overview of strategies to modulate protease activity or expression includes a variety of approaches such as gene silencing, protease absorption, inhibition through metal ion cofactor chelation, induction of conformational changes, and competitive inhibition has been shown in Figure 3. Additionally, any intervention that promotes wound healing—such as resolving local infections or managing systemic and local disease conditions—can indirectly contribute to the regulation of protease activity.

Recent advancements have focused on delivering siRNA to specifically downregulate MMP‐9 expression. A notable study developed a biodegradable nanofiber dressing embedded with a nanosystem composed of cationic polymer‐coated fluorescent nanodiamonds carrying siRNA against MMP‐9. This dressing facilitated the controlled release of siRNA into the wound environment. In a diabetic murine model, application of this dressing led to a significant reduction in MMP‐9 mRNA and protein levels, resulting in accelerated wound closure and scar formation comparable to nondiabetic controls. These findings underscore the potential of siRNA‐based therapies in restoring the balance of protease activity in chronic wounds [262]. Another innovative approach involved the incorporation of siRNA targeting Fidgetin‐like 2, a negative regulator of cell migration, into a surfactant polymer dressing (SPD). This combination enhanced keratinocyte and fibroblast motility, leading to improved wound healing outcomes in mice. The SPD facilitated localized and sustained delivery of siRNA, highlighting the importance of effective delivery systems in gene silencing therapies [263].

Other than that protease absorption and inhibition through metal ion cofactor chelation strategies have explored for modulating protease activity within chronic wound environments. Hydrogels functionalized with protease‐binding agents have shown promise in sequestering excessive proteases from wound exudates. For instance, bisphosphonate‐functionalized hydrogels have been developed to attenuate MMP activity in chronic wound fluid. These hydrogels incorporate alendronate, a bisphosphonate known for its high affinity to divalent metal ions, which are essential cofactors for MMP activity. By binding to these metal ions, the hydrogel effectively inhibits MMPs, reducing proteolytic degradation within the wound bed. Importantly, these hydrogels have demonstrated biological inertness in ex vivo human skin models, suggesting their safety and potential efficacy in clinical applications [264]. Numerous commercial wound care products have been developed to modulate protease levels, either by direct absorption, enzymatic inhibition, or altering the wound environment. This section summarizes key findings on how various dressings, gels, and biomaterials influence protease concentration and activity, with particular emphasis on MMP‐2, MMP‐9, and elastase. Cullen et al. developed a sponge composed of freeze‐dried bovine collagen and oxidized regenerated cellulose (ORC), analogous to Promogran, and demonstrated its ability to significantly reduce elastase‐like and plasmin‐like activity in diabetic wound exudates, though gelatinolytic activity remained high, likely due to limited sample size and persistent gelatin degradation [265]. Lobmann et al. evaluated Promogran in DFUs and found no significant changes in MMP concentrations or mRNA levels, but a reduced MMP‐9/TIMP‐2 ratio and a 14% greater wound area reduction were noted [266].

Another study with daily Promogran treatment reported no tissue‐level differences in MMP expression but noted reduced MMP‐2 activity in wound exudates [251]. Promogran Plus (with silver) showed minimal effect on elastase and MMP‐2 levels after 4 weeks. In PU patients, combining Promogran with foam dressing (TIELLE) led to lower elastase activity compared with TIELLE alone. Additionally, Issa et al. demonstrated that Promogran and Promogran Prisma significantly enhanced wound closure and reduced protease activity in in vitro tissue models exposed to bacterial supernatants [267]. Metzmacher et al. [268] found that both Promogran and Suprasorb C (collagen sponge) comparably absorbed MMP‐2, MMP‐9, and bacterial collagenase. Schönfelder et al. [269] further reported that Suprasorb C was more effective than Promogran in absorbing polymorphonuclear elastase, with Tabotamp showing the highest efficacy and bacterial cellulose the least. These results, based on 0.5 cm^2^ samples, likely reflect differences in thickness and density, not just chemical composition. Similar trends were seen in chronic wound fluid tests, where Tabotamp completely absorbed elastase. Smeets et al. [270] demonstrated that combining Promogran with a hydrocolloid dressing reduced elastase activity more than the hydrocolloid alone, though no differences were observed for MMP‐2 or plasmin. Cutimed Epiona matched Promogran in MMP‐9 absorption but was less effective for MMP‐2, while Endoform showed the lowest sorption overall. However, water‐soluble compounds from Endoform inhibited MMPs and NE more effectively than Promogran extracts, though it was less effective in direct MMP‐9 inhibition. Another study reported that Suprasorb A, Suprasorb A+Ag, and Acticoat Absorbent significantly absorbed elastase [271]. Additionally, Rodriguez et al. found that macrophages cultured on Manuka honey‐based dressings, including APIS and a honey–alginate composite, secreted less MMP‐9 than those cultured on collagen dressings or standard plates [251]. Superabsorbent dressings demonstrated protease absorption capabilities: Mextra and Eclypse absorbed MMP‐2 and ‐9 (up to 50 and 20%, respectively), while Sorbion Sachet EXTRA fully suppressed collagenase activity and reduced MMP and elastase levels. Tegaderm Superabsorber outperformed Zetuvit in absorbing MMP‐1, ‐2, and elastase, with both absorbing ∼90% of MMP‐9. Aquacell Foam also sequestered MMP‐2 and ‐9. Oasis, a porcine small intestine submucosa matrix, slightly inhibited MMP‐1 in vitro, but not MMP‐2 or ‐9. In vivo studies showed variable results: minor early reductions in MMPs in diabetic mice and significantly less increase in MMPs in responsive VLUs compared with nonresponders, though overall levels remained unchanged. Kerralite Cool, a hydrogel, inhibited MMP‐2 and ‐9 activity in zymogram‐based assays. GBT013, a freeze‐dried matrix of collagen, chitosan, and chondroitin sulfate, reduced MMP‐9 and ‐2 activity by up to 89 and 53%, respectively, and upregulated TIMP‐1 and ‐2 expression in fibroblasts. Conversely, PluroGel and PluroGel PSSD increased MMP‐2 and ‐9 activity (up to 200%) while reducing MMP‐8, with no clear explanation for the differential effects. Ashcroft et al. [272] showed that an estrogen patch (Evorel) reduced elastase activity in biopsies from elderly individuals. Lamin gel, a glycyl‐l‐histidyl‐l‐lysine copper–tripeptide complex, reduced MMP‐2 and ‐9 levels in ischemic wounds in rats [273]. Flaminal Forte, a gel with debriding and antibacterial properties, also inhibited MMP‐2 and ‐9 activity, likely due to oxidative enzyme‐generated radicals [274].

While elevated protease activity is typically associated with impaired wound healing, paradoxically, exogenous proteases have also been investigated and applied therapeutically in certain contexts. One prominent application is in biofilm disruption, which enhances antibiotic penetration. For instance, investigators utilized Ficin, a cysteine protease from Ficus latex, and further immobilized it on chitosan to improve its stability against hydrolysis and autolysis [275]. Proteases are also clinically employed for enzymatic debridement, facilitating the removal of necrotic tissue and eschar to prepare the wound bed for healing. Commercially available protease‐based products include Santyl (collagenase), NexoBrid, Bionect Start, Accuzyme, and Novuxol. Another proposed mechanism is the selective degradation of detrimental endogenous proteases. For example, Gao et al. [276] observed elevated MMP‐9 in diabetic wounds but not MMP‐8, suggesting a differential role where MMP‐9 impairs and MMP‐8 supports healing. They demonstrated marginal wound closure improvement by supplementing MMP‐8, with greater benefit when combined with an MMP‐9 inhibitor [276]. Botanical proteases have also shown promising wound healing effects, although mechanisms remain partly unclear. Kulkarni et al. [277] reported enhanced wound closure in mice treated with a protease from *Maclura spinosa*, an effect negated by protease inhibition. Similar healing benefits were observed with proteolytic extracts from *Wrightia tinctoria*, *Plumeria rubra*, and *Vasconcellea cundinamarcensis*, some of which also exhibited anti‐inflammatory properties [278]. In summary, while endogenous proteases in chronic wounds are often pathologic, controlled application of exogenous proteases—particularly from plant sources—can aid wound management through biofilm disruption, debridement, and potentially modulating local inflammation or protease profiles. Taken together, these protease‐targeted strategies underscore the dual role of proteases in both immune dysregulation and tissue breakdown in chronic wounds. By modulating their activity, these therapies can reset the inflammatory milieu, promote immune cell functionality, and facilitate tissue regeneration. However, despite encouraging preclinical and early clinical data, further large‐scale trials are needed to optimize dosing, delivery mechanisms, and patient‐specific applications to fully realize their translational potential. These findings support the selective and context‐dependent use of proteases as therapeutic agents in chronic wound care.

### 5.4 Stem Cells‐Based Therapies

MSCs have emerged as a pivotal component in the therapeutic landscape for chronic wound healing due to their unique regenerative properties, immunomodulatory effects, and ability to promote tissue repair. Their ability to differentiate into various cell types, secrete bioactive factors, and interact with other cell populations positions MSCs as an innovative solution for managing chronic wounds, such as diabetic ulcers, VLUs, and pressure sores. The application of MSCs in treating chronic skin wounds is a rapidly evolving area of research that harnesses their ability to modulate immune responses. Various studies have conducted to explore the therapeutic efficacy of MSCs in promoting wound healing through multiple mechanisms, including enhanced cellular communication, secretion of growth factors, and modulation of inflammation [279, 280, 281]. MSCs exert immunomodulatory effects through multiple mechanisms, including direct cell‐to‐cell interactions—such as PD‐L1 and Fas Ligand binding to T‐cells—and the secretion of various soluble factors like indoleamine 2,3‐dioxygenase (IDO), TGF‐β1, IL‐10, PGE2, hepatocyte growth factor (HGF), galectins‐1 and ‐9 (GAL‐1, GAL‐9), NO, and IL‐1 receptor antagonist (IL‐1Ra), as well as the release of EVs. Collectively, these mechanisms contribute to a wide range of immunoregulatory outcomes, including the promotion of M2 macrophage polarization, inhibition of mast cell degranulation, suppression of NK cell proliferation and cytotoxicity, reduction of polymorphonuclear neutrophil (PMN) activity, inhibition of DC maturation, and induction of Treg cells and Th2 CD4+ T‐cells subsets. Overall, MSCs modify the inflammatory behavior of macrophages [282], promote the generation of new blood vessels, enhance angiogenesis [283], and contribute to the formation of granulation tissues, skin cells, and ECM production [284].

The multistage process of MSCs immunomodulation includes (1) MSCs response to inflammation and subsequent migration to injury site, (2) activation of MSCs, (3) facilitation of pathogen clearance if necessary, and (4) immunomodulation. A developing theory in the MSCs field holds that MSCs do not naturally inhibit or promote the immune system; rather, they need activation to insert immunomodulation. The activation of MSCs to modify immune responses has been shown to depend on IFN‐γ, TNF‐α, IDO, IL‐1β, IL‐6, and IL‐10 signaling as well as proteins and RNAs that control macrophages differentiation [285]. Recently, two very intriguing concepts have been put forth, according to which: (1) MSCs have monitoring functions that allow them to sense their microenvironment and respond appropriately; and (2) MSCs become polarized toward either an immunosuppressive phenotype or a proinflammatory phenotype depending on the TLR signals received [285]. Together, these concepts serve to clarify some of the ambiguous evidence indicating that MSCs sometimes promote immune cell survival and function while other times they suppress inflammation and promote repair. TLR2/4 priming of MSCs generated a proinflammatory phenotype and caused the production of IL‐6, IL‐8, and TGF‐β, which was allegedly increased by costimulation with IFN‐γ. TLR3 stimulation, in contrast, caused MSCs to produce anti‐inflammatory molecules such IDO, PGE‐2, IL‐4, and IL‐1RA [286].

MSCs exhibit remarkable adaptability, allowing them to respond effectively to varying microenvironments, facilitating tissue repair and regeneration. While TLR sensing is a recognized mechanism through which MSCs recognize environmental cues, there are several other intricate mechanisms by which MSCs sense and respond to their surroundings. Their ability to sense and respond to ECM properties, inflammation status, hypoxic conditions, as well as to engage in cell–cell interactions and communicate through exosomes, underscores their versatility in tissue repair and regeneration. The ability of MSCs to respond to ECM stiffness, composition, and architecture is critical. It has been shown that the stiffness of the ECM can direct MSC lineage specification and modulate their secretory functions [287, 288]. Specifically, MSCs cultured on substrates with varying stiffness levels exhibit differential secretion of bioactive factors. For instance, the secretion of VEGF, a key proangiogenic factor, is maximized when MSCs are cultured on hydrogel matrices with a stiffness of approximately 5.0 kPa. This suggests that an optimal mechanical environment can enhance the therapeutic potential of MSCs by promoting angiogenesis through increased VEGF secretion [287]. Beyond stiffness, specific ECM components influence MSC behavior through mechanotransduction pathways. For example, cadherins, which are cell adhesion molecules, act as force sensors that can activate cytoskeletal remodeling and signal transduction in response to mechanical cues. MSCs cultured on full‐length N‐cadherin extracellular domains (EC1–5) demonstrate stiffness‐dependent changes and significantly higher secretion of VEGF and IGF‐1. Additionally, laminin, another ECM protein, enhances the secretion of growth‐regulated oncogene‐alpha (GRO‐α/IL‐8) and HGF in placental‐derived stem cells through the JNK and PI3K/AKT signaling pathways, respectively, which contributes to reduced reactive oxygen species production and cardiomyocyte apoptosis [287]. This mechanosensitive behavior allows MSCs to adapt their functional outputs, including paracrine signaling and differentiation, based on ECM characteristics, thereby promoting an optimal healing response. These findings underscore the importance of the mechanical properties of the cellular microenvironment in regulating MSC function. By tailoring ECM stiffness and composition, it is possible to enhance the paracrine effects of MSCs, thereby improving their efficacy in tissue regeneration and repair. This has significant implications for the design of biomaterials and scaffolds in regenerative medicine applications.

MSCs have been demonstrated to exert their immunosuppressive impact remotely through paracrine function, which releases a range of soluble substances with antiapoptotic, anti‐inflammatory, angiogenic, and immunomodulatory properties. In scenarios lacking direct cell–cell contact, the distance between MSCs and target cells affects the mode of paracrine signaling. When cells are farther apart, MSC‐EVs become the primary mediators of intercellular communication. For example, in coculture systems of MSCs and human umbilical vein ECs (HUVECs), increased intercellular distances (greater than 400 µm) lead to elevated levels of VEGF, FGF‐2, and integrin subunit alpha 3 within MSC‐EVs. This suggests that greater distances enhance the proangiogenic potential of MSCs through EV‐mediated signaling [287]. Human periodontal ligament‐derived MSCs (hPDL‐MSCs) have been shown to suppress CD4⁺ T lymphocyte proliferation and cytokine secretion even in indirect coculture systems, where direct contact is prevented. This suppression is mediated by soluble factors such as IDO‐1, PGE‐2, and TNF‐stimulated gene‐6 (TSG‐6). Although the immunosuppressive effect is more pronounced with direct contact, significant modulation occurs through paracrine signaling alone [289]. Moreover, MSCs modulate adaptive immunity mainly through paracrine signaling. They suppress Th17 cell differentiation by secreting IL‐10, PGE2, and influencing histone modifications, thereby reducing proinflammatory cytokines like TNF‐α, IL‐17, IL‐22, and IFN‐γ. The effect varies with cytokine priming and MSC/T‐cells ratios. While the mechanisms are not fully understood, IL‐25 knockdown study highlight the role of the IL‐25/STAT3/PD‐L1 axis as a potential therapeutic target [290]. Additionally, MSC‐derived IDO promotes Treg cell formation and transplant tolerance, and PD‐L1/PD‐L2 inhibit IL‐2 release and CD4⁺ T‐cells activation, inducing immune suppression and peripheral tolerance [291]. MSCs control adaptive immune responses by secreting PGE‐2 which causes DCs to secrete more of the anti‐inflammatory cytokine such as IL‐10 and less of the proinflammatory cytokines such as IL‐12 and TNF‐α. As a result, proinflammatory Th1 cells transform into anti‐inflammatory Th2 cell phenotypes. Concurrently, naive T‐cells undergo differentiation into Treg cells, resulting in an additional reduction of Th cells overall [292].

Several in vivo studies have shown that application of MSCs may induce significant changes in the inflammation resolution and wound repair (Table 3).

**TABLE 3:** MSCs‐based therapies to promote skin regeneration and wound healing in animal models.

Treatment	Model	Condition	Result	References
FD‐MSCs	Mouse	Acute skin wound model	Significantly higher expression of arginase‐1 indicated an enriched M2 macrophage environment, along with faster wound healing, enhanced collagen deposition, and improved vascular regeneration compared with controls.	[293]
BM‐MSC‐CM	Mouse	Full‐thickness skin wounds	Increased neutrophil and macrophage infiltration enhanced granulation tissue formation and resolution, increased vasculature and regeneration of hair follicles in acute wounds. Resolution of granulation tissue formation and increased infiltration of prohealing M2 macrophages in chronic wounds.	[294]
BM‐MSCs	Mouse	Excisional wound	Enhanced wound healing and collagen fibers	[295]
	Mouse	Diabetic model	BM‐MSC‐loaded scaffolds accelerated wound closure by improving collagen deposition, angiogenesis, and re‐epithelialization. RNA sequencing revealed upregulation of genes, associated with skin regeneration and inflammatory modulation.	[296]
	Mouse	Diabetic model	Hypoxic BM‐MSCs exhibited increased secretion of VEGF.	[297]
	Mouse and canine	Excisional wound	Collagen pretreatment enhanced BM‐MSC proliferation and secretion of growth factors and chemokines associated with skin repair.	[298]
UC‐MSCs	Mouse	Diabetic model	By introducing the IL‐4, IL‐10, and IL‐13 genes into UC‐MSCs, the resulting MSCs‐3IL exhibited strong expression of anti‐inflammatory factors and improved wound healing and promoted M2 polarization compared with unmodified UC‐MSCs.	[279]
	Rat	Cold burn wound model	Enhanced regeneration of skin layers along with hair follicles, increased neovascularization. Gene profile of wound healing mediators illustrated significant upregulation of IL‐5, IL‐4, GPX‐7, TXNRD‐2, PRDX, VEGF, and FGF and downregulation of inflammatory cytokines IL‐1β and IL‐6.	[299]
WJ‐MSCs	Mouse	Excisional wound	Enhancing usual skin fibroblast growth	[295]
WJ‐MSCs primed by Poly I:C or IFN‐γ	Mouse	Atopic dermatitis (AD)	Reduction in inflammatory cell infiltration and epidermal thickness in skin lesions	[300]
AMSCs	Mouse	Diabetic skin wound	Promotion of angiogenesis	[301]
MSCs seeded on fibrin hydrogels	Mouse	Excisional wound	Enhancing angiogenesis by inducing the growth of ECs, and stimulating macrophage polarization	[180]
BM‐MSCs seeded on biocompatible hydrogel	Rat	Diabetic foot ulcers (DFU)	M1 macrophage activity inhibition, M2 macrophage stimulation, induction of granulation tissue development, and angiogenesis	[302]
BM‐MSCs seeded on the artificial dermal matrix (ADM)	Rat	Excisional wound	Promoting reepithelization, neo‐angiogenesis, and reducing collagen synthesis to enhance skin regeneration	[303]
Hypoxia‐preconditioned MSCs loaded onto Integra	Mouse	Excisional wound	Decreased proinflammatory cytokines, including IL‐6 and IL‐1β, along with a reduced infiltration of inflammatory cells, such as PMNs and M1 macrophages	[189]
MSC spheroids loaded onto HA hydrogels	Mouse	Full‐thickness skin defect model	Reduction of the concentrations of proinflammatory proteins such as TNF‐α and high mobility group box 1 (HMGB1)	[304]
AT‐MSCs seeded onto hydrogels of PEG macromers & thiolated hyaluronic acid	Rat	Diabetic wound	Reduced the expression of inflammatory factors such as CD11b, TNF‐α, and IL‐1β	[189]

Abbreviations: AF, amniotic fluid; AMSCs, amniotic mesenchymal stromal cells; AT, adipose tissue; BM‐MSC‐CM, bone marrow‐MSC‐derived conditioned medium; BM, bone marrow; MSCs, mesenchymal stromal cells; PMNs, polymorphonuclear neutrophils; Poly I:C, polyinosinic:polycytidylic acid; UC, umbilical cord; WJ, Wharton's jelly; FD‐MSCs, fetal dermal mesenchymal stem cells.

As highlighted throughout this section, MSCs hold considerable promise for promoting wound healing and skin regeneration due to their unique combination of multipotent differentiation capacity and paracrine activity, including the secretion of anti‐inflammatory, proangiogenic, and immunomodulatory mediators. However, the translation of MSC‐based therapies into clinical practice remains challenging by substantial variability in delivery protocols and dosing regimens across studies. These inconsistencies hinder the ability to clearly define the influence of timing, route of administration, delivery systems, and cell dosage on therapeutic outcomes and engraftment efficiency. Emerging as a promising cell‐free alternative, MSC‐derived exosomes have attracted considerable attention for their ability to recapitulate many of the therapeutic effects of parent MSCs, while circumventing concerns related to cell viability, immune compatibility, and tumorigenicity. Nevertheless, scalability remains a major bottleneck, as current methods yield insufficient quantities of exosomes for widespread clinical use. Overall, the analysis of dynamic response by MSCs to their microenvironment, positions them as a promising therapeutic option for chronic wound healing. However, further research is needed to optimize their clinical application, including understanding their interactions with immune cells, ECM components, and microbial communities.

### 5.5 EVs‐Based Therapies

EVs are membrane‐bound structures that transport genetic material, proteins, and lipids between cells or integrate into the ECM. Practically, EVs are categorized by size into large EVs (>200 nm) and small EVs (<200 nm), encompassing various subtypes such as exophers, apoptotic bodies, ectosomes, and exosomes. While EVs have been studied across multiple cell types, immune cell‐derived and MSC‐derived EVs (MSC‐EVs) have emerged as key tools for achieving immunomodulatory effects. Additionally, the development of engineered or modified EVs with enhanced biochemical properties has gained increasing attention for therapeutic applications. Over the past decade, a major focus of EVs research has been on utilizing MSC‐EVs to modulate immune responses in chronic wound settings. MSCs are well‐recognized for their ability to regulate both innate and adaptive immunity. Notably, beyond direct cell‐to‐cell contact and the release of soluble factors, numerous studies have demonstrated that MSC‐EVs significantly promote macrophage polarization toward the M2 phenotype, contributing to improved wound healing outcomes [305, 306].

During wound healing, MSCs can secrete EVs rich in miRNAs that influence the regenerative capabilities of adjacent cells. These secreted signals are crucial for coordinating the local healing process and tailoring the inflammatory response, thereby ensuring efficient healing while mitigating excessive inflammation. For example, engineered BMSC‐derived exosomes enriched with miR‐542‐3p have been reported to enhance the proliferation, migration, and angiogenesis of human skin fibroblasts and dermal microvascular ECs [307]. Moreover, MSC‐derived exosomes transfer miR‐223 to macrophages, increasing its intracellular levels. miR‐223 targets and downregulates the transcription factor Pknox1, a known inhibitor of M2 polarization. This downregulation facilitates the shift of macrophages from the M1 phenotype to the anti‐inflammatory M2 phenotype [308]. M2 macrophages are characterized by elevated expression of markers such as CD206, Arginase‐1, and RELM‐α, which are associated with tissue repair and anti‐inflammatory functions [189]. Researchers revealed that the enhancement of macrophage phagocytic activity by MSCs is closely linked to the uptake of MSC‐EVs containing MSCs’ mitochondria [309]. Growing evidence underscores the significant role of MSC‐EVs in regulating the balance between M1 and M2 macrophages, though the precise mechanisms remain to be fully elucidated. Additionally, MSCs are known to release C–C chemokine ligand 2 (CCL2, also known as MCP‐1), which attracts monocytes and macrophages to the site of injury, thereby promoting wound healing [309]. EVs from human umbilical cord MSCs (UCMSCs) have been shown to induce M2 polarization and increase IL‐10 expression via the PI3K–Akt pathway [310]. Preconditioning MSCs alters the cargo of their EVs, enriching them with specific molecules. For example, EVs from LPS‐treated MSCs promote M2 macrophage polarization through miRNA‐let‐7b and activation of the TLR4/NF‐κB/STAT3/AK pathway. Similarly, EVs enriched with miRNA‐181c or miRNA‐182 activate this pathway, suppressing proinflammatory cytokines like TNF‐α and IL‐1β, while enhancing anti‐inflammatory mediators such as TGF‐β and IL‐10. Other preconditioning agents—like TNF‐α, IFN‐γ, melatonin, NO, and hypoxia—have also enhanced the ability of MSC‐EVs to induce M2 macrophage polarization [189]. Beyond macrophages, MSC‐EVs have shown regulatory effects on Th cells, cytotoxic CD8+ T‐cells, B cells, NK cells, and APCs. They have been reported to increase IL‐10 levels and reduce activation of Th1, Th17, and DCs in vivo, as well as promote Treg cell induction from naïve CD4+ T‐cells in vitro. However, in vivo effects on Treg cells remain inconsistent suggesting that the complexity of the in vivo environment may limit direct translation from in vitro results [189]. Evidence indicates that MSC‐EVs can promote the conversion of activated T‐cells into Treg cells, thereby suppressing inflammatory reactions. The in vitro and in vivo studies have demonstrated the ability of MSC‐EVs to induce this immunomodulatory effect. For instance, in vivo injection of MSC‐EVs has been shown to suppress the immune response of cytotoxic T‐cells and Th1, reduce the levels of proinflammatory cytokines such as TNF‐α and IFN‐γ, and promote the induction of Treg cells and the anti‐inflammatory cytokine IL‐10, thereby preventing the onset of allergic contact dermatitis, a typical T‐cells‐mediated disease, in a mouse model [309, 311]. This effect was further confirmed through in vitro studies, where MSC‐EVs influenced the metabolism of Th1‐type differentiated T‐cells via the TGF‐β signaling pathway [312]. Additionally, in further in vivo experiments, immune cells predominantly took up MSC‐EVs derived from biofunctional scaffolds. These scaffolds and exosomes worked in tandem, with scaffolds acting as immune cell recruiters and MSC‐EVs as trainers, collectively enhancing Treg cell responses in a mouse model of skin injury [309, 313]. Thus, the selective packaging and delivery of miRNAs by MSCs enable them to fine‐tune their interactions within the tissue environment, exerting targeted effects that promote healing.

MSC‐EVs have demonstrated significant potential in promoting wound healing in diabetic skin by reducing inflammation and enhancing macrophage polarization. In a LPS‐induced wound model, human exfoliated deciduous teeth (SHED)‐EVs were shown to boost macrophage autophagy through activation of the AKT, ERK1/2, and STAT3 signaling pathways, thereby promoting wound healing and alleviating itching [314]. Additionally, melatonin‐pretreated MSC‐EVs were found to inhibit the proinflammatory cytokines IL‐1β and TNF‐α, activate the PTEN/AKT signaling pathway, increase the M2/M1 polarization ratio, and suppress the inflammatory response, thus facilitating diabetic wound healing [314]. Other than MSC‐EVs, keratinocyte‐derived EVs play a significant role in modulating immune responses during skin inflammation [315]. Studies have demonstrated that cytokine‐treated keratinocyte‐derived EVs can significantly induce neutrophil production and release, as well as the expression of proinflammatory cytokines such as IL‐6, IL‐8, and TNF‐α in neutrophils. This induction occurs through the activation of key signaling pathways, including NF‐κB and p38 MAPK pathways [316]. Additionally, these EVs have been shown to promote NET formation, a process that contributes to the containment of pathogens but can also exacerbate tissue damage in chronic inflammatory conditions. Furthermore, keratinocyte‐derived EVs are recognized as key regulators of macrophage trafficking and the maintenance of the epithelial barrier following injury [309]. Levy et al. [317] identified the proangiogenic and anti‐inflammatory properties of induced pluripotent stem cell (iPSC)‐derived MSC‐EVs in a mouse model of diabetic wound healing. These EVs effectively promote inflammation resolution within the wound bed, supporting the healing process [317]. The findings highlight the potential of iPSC‐derived MSC‐EVs as a therapeutic approach for enhancing wound repair in diabetic conditions through their immunomodulatory effects.

EVs derived from various immune cell types have been extensively studied for their role in regulating immune responses. For instance, DC‐derived EVs (DC‐EVs) are enriched with MHC class I and II molecules, heat shock proteins, adhesion molecules, and costimulatory factors, all of which contribute to the activation and modulation of immune cells. Similarly, EVs released by activated T‐cells carry transfer RNA fragments that enhance T‐cells activation. Additionally, macrophage‐derived EVs play a crucial role during the inflammatory phase by facilitating the transition from M1 to M2 macrophage phenotypes, supporting immune resolution and tissue repair [318]. Innovative approaches have been employed to enhance the immunomodulatory effects of MSC‐EVs. Zhao et al. [319] developed genetically engineered UCMSC‐derived exosomes enriched with endothelial NOS (eNOS). These engineered exosomes effectively remodeled the immune microenvironment in diabetic wounds by modulating neutrophil infiltration, promoting M2 macrophage polarization, and increasing Treg cell populations, leading to improved tissue repair. Moreover, Wang et al. [320] investigated the role of apoptotic MSC‐derived EVs in cutaneous wound healing in type 2 diabetic mice. Their findings revealed that these EVs ameliorated wound healing by inhibiting macrophage pyroptosis, a form of programmed cell death associated with inflammation. This inhibition led to reduced inflammatory responses and enhanced tissue regeneration. Park et al. [256] explored the therapeutic effects of serpin‐loaded EVs in a mouse model of impaired wound healing. The study demonstrated that these EVs promoted tissue repair by modulating the inflammatory response and enhancing ECM remodeling, highlighting the potential of cargo‐loaded EVs in regenerative medicine. Researchers have engineered exosomes derived from UCMSCs by loading them with eNOS using optogenetic techniques [319]. These modified exosomes demonstrated superior therapeutic effects in diabetic wound models by suppressing inflammation, promoting angiogenesis, and enhancing tissue repair. These studies collectively emphasize the promising role of MSC‐EVs, both native and engineered, in modulating immune responses to facilitate chronic skin wound healing. Several in vivo studies have shown that application of EVs may induce significant changes in the inflammation resolution (Table 4).

**TABLE 4:** EV‐based therapies to promote immunomodulation of chronic wound healing in animal models.

Treatment	Model	Condition	Result	References
MSCs‐EVs‐HOTAIR	Mouse	Diabetic (db/db) mice	Promoted angiogenesis and wound healing in diabetic (db/db) mice along with upregulation of VEGF in ECs	[321]
MSCs‐EVs‐HMOX1	Mouse	Diabetic wound model mice	Facilitated faster wound healing, re‐epithelialization, collagen deposition, and angiogenesis	[322]
BMSCs‐EVs	Rat	Diabetic wound	Accelerated wound closure, re‐epithelization, collagen deposition, and neovascularization, and reduced wound inflammation	[323]
	Mouse	DFU	Exosomal circ‐ITCH from BMSCs inhibited ferroptosis and improved the angiogenesis of HUVECs through activation of the Nrf2 signaling pathway by recruiting TAF15 protein, ultimately accelerating the wound healing process	[324]
BMSC‐EVs‐derived lncRNA KLF3‐AS1	Mouse	Diabetic cutaneous wound model	Increased blood vessel formation, reduced inflammation, decreased miR‐383 expression, and upregulated VEGF‐A	[325]
AT‐EVs	Mouse	DFU	Exosomal mmu_circ_0001052 inhibited apoptosis and miR‐106a‐5p expression, and meanwhile promoted proliferation, migration, angiogenesis and expressions of FGF4, VEGF, and p‐p38/p38.	[326]
	Mouse	Diabetic cutaneous wound model	Diabetic AT‐EVs stimulated resident monocytes/macrophages to secrete more TGF‐β1 and activate the TGF‐β/Smad3 signaling pathway, stimulated wound healing by dermal cell proliferation, keratinocyte proliferation, and angiogenesis.	[327]
	Mouse	Delayed wound healing model of diabetes	Enhanced skin collagen production, angiogenesis, cell proliferation, inhibited apoptosis, promoted skin barrier function repair, and reduced inflammation in skin lesions	[328]
	Mouse	Full‐thickness skin wound on diabetic (db/db) mice	Improved the level of high‐glucose‐induced oxidative stress, promoted angiogenesis, and reduced mitochondrial functional impairment and the inflammatory response by regulating SIRT3/SOD2, thus promoting diabetic wound healing	[329]
AT‐EVs loaded with miR‐21‐5p	Rat	Full‐thickness skin defects in diabetic rats	Accelerated diabetic wound healing by increasing re‐epithelialization, collagen remodeling, angiogenesis, and vessel maturation	[330]
GMSCs‐EVs	Rat	Diabetic rat skin defect model	Promoted healing of diabetic skin defects with more neo‐epithelium and collagen in the hydrogel‐exosome group along with the highest microvessel density and nerve density	[256]
UC‐EVs	Mouse	STZ‐induced diabetic model	Improved wound healing, granulation tissue formation, blood perfusion	[331]
UC‐EVs/eNOS	Mouse	Diabetic wound	Improved the inflammatory profile at the wound site and modulated the associated immune microenvironment, thus significantly promoted tissue repair, improved the rate of wound closure and enhanced vascular neogenesis and matrix remodeling in diabetic mice	[319]
UC‐EVs loaded with miR‐21‐5p	Mouse	Full‐thickness cutaneous wounds on diabetic mice	Increased the capillary density and elevated expression of angiogenic markers such as CD31 and VEGF. MiR‐17‐5p activated the AKT/HIF‐1α/VEGF signaling pathway, thereby promoted angiogenesis and accelerated the wound healing	[332]
SHED‐EVs	Mouse	Diabetic wound	SHED treated MSC‐EVs inhibited proinflammatory cytokines IL‐1β and TNF‐α, activated the PTEN/AKT signaling pathway, and increased the M2/M1 polarization ratio.	[314]
iPSC‐MSC‐EVs	Mouse	Diabetic wound	iPSC‐EVs more effectively mediated inflammation resolution within the wound bed.	[317]
MSC‐EVs immobilized on biofunctional scaffolds	Mouse	Excisional wound	Enhanced Treg cell responses	[314]
GelMA‐EVs hydrogels	Mouse	Subcutaneous pockets	Promoted macrophage polarization toward an M2 phenotype	[333]
MSC‐EVs seeded onto gellan gum‐HA hydrogels	Mouse	Type 1 diabetes	Increased M2/M1 macrophage ratio in a diabetic mouse wound model	[189]
MSC‐EVs	Mouse	Type 2 diabetes	Inhibited macrophage pyroptosis which led to reduced inflammatory responses	[320]
AECs‐EVs	Rat	Skin injury	Enhanced fibroblast migration and proliferation, and downregulated collagen expression	[285]
HF‐MSCs‐EVs	Mouse	Diabetic skin wound	Inhibited pyroptosis by reversing the stimulation of the NLRP3 inflammasome	[307]

Abbreviations: AECs, amniotic epithelial cells; AT, adipose tissue; BM, bone marrow; EVs, extracellular vesicles; GMSCs, gingival mesenchymal stem cells; HA, hyaluronic acid; HF‐MSCs, hair follicle‐derived MSCs;HOTAIR, noncoding RNA HOX transcript antisense RNA; iPSC, induced pluripotent stem cells; miRNAs, microRNAs; MSCs, mesenchymal stromal cells; UC, umbilical cord.

Although MSC‐EVs and EVs from other cell types possess intrinsic immunomodulatory capabilities, their unique biomechanical and physicochemical properties—such as nanoscale size, deformability, low immunogenicity, and hydrophobic surfaces with hydrophilic cores—make them highly promising carriers for advanced therapeutic strategies. Researchers have enhanced the therapeutic efficacy of EVs by engineering their cargo with bioactive compounds, including small molecules, proteins, nucleic acids, and nanoparticles. For instance, loading macrophages‐derived EVs with curcumin has been shown to boost their anti‐inflammatory activity [334]. Similarly, the overexpression of therapeutic proteins like TSG‐6 within MSC‐EVs has been linked to reduced inflammation and minimized scar formation in vivo [335]. Among nucleic acid‐based modifications, miRNAs have gained particular attention in chronic wound healing. Engineered EVs enriched with miR‐126, miR‐31‐5p, or miR‐146a have demonstrated improved wound closure and tissue regeneration in preclinical models [336]. In clinical research, a few trials involving unmodified MSC‐derived EVs have reached early‐phase stages, such as those addressing T1D mellitus (phase II/III) [NCT02138331] and burn wound treatment (phase I) [NCT05078385] [336] (see Table 5). These studies underscore the therapeutic potential of EVs and the importance of overcoming current analytical and regulatory challenges to accelerate their clinical translation. This number highlights the significant translational barriers that engineered EVs still face on the path to clinical implementation. Despite these limitations, EVs are emerging as highly adaptable and innovative therapeutic tools. Current approaches using unmodified, source‐specific EVs—considered first‐generation—are now being complemented by the development of second‐generation engineered EVs with enhanced functionality.

**TABLE 5:** Summary of clinical trial‐based therapies for chronic wound healing: biological and nonbiological products used to date.

Treatment	Composition	Target disease	Clinical phase	Status	Results	NCT
MSC‐EV	EVs	Diabetes mellitus type 1	II/III	Unknown status	Not reported	NCT02138331
MSC‐EV	EVs	Burn wounds	I	Not yet recruiting	Not reported	NCT05078385
PEP (purified exosome product)/TISSEEL	PEP drug product delivered in 10 mL TISSEEL fibrin sealant. PEP drug product is a lyophilized powder (EVs) derived from apheresed platelets in plasma.	DFUs	IIa	Recruiting	Not available	NCT06319287
Extracellular micro vesicles	Extracellular micro vesicles (EV) as direct perilesional injection into the diabetic chronic foot ulcers (DFU)	DFUs	I	Active	Not available	NCT06825884
SER‐VES‐HEAL	Autologous serum‐derived EV	Venous trophic lesions	Not applicable	Unknown	Not reported	NCT04652531
PEP on a skin graft donor site wound	Platelet‐derived EVs enriched in anti‐inflammatory and angiogenic growth factors	Skin graft donor site wound	I	Active, not recruiting	Not available	NCT04664738
MSC EVs	EVs	Epidermolysis bullosa	I/II	Recruiting	Not available	NCT04173650
Filsuvez	A topical gel containing 10% birch bark extract in 90% sunflower oil	Epidermolysis bullosa	III	Completed	Modulated inflammatory mediators	NCT03068780
InnovaMatrix AC	Porcine placental ECM	Wound heal, VLUs	Not applicable	Recruiting	Not available	NCT06606210
InnovaMatrix AC	Porcine placental ECM	Chronic VLUs		Completed	Not reported	NCT06400875
MatriStem + Mepilex	MatriStem [urinary bladder matrix] + Mepilex [Silicone foam dressing]	Neuropathic DFUs	Not applicable	Completed	Not reported	NCT02750280
PREPARE	Purified native type 1 collagen ECM with polyhexamethylene biguanide antimicrobial (PCMP)	Nonhealing DFUs	Not applicable	Recruiting	Not available	NCT06618612
NPWT combined with type‐I collagen‐based advanced skin substitute	High purity type‐I collagen‐based skin substitute + negative pressure wound therapy	Full thickness skin defects, ulcer	Not applicable	Not yet recruiting	Not available	NCT06873867
HPTC Vs dHACM	High‐purity type‐I collagen‐based skin substitute (HPTC) and dehydrated human amnion/chorion membrane (dHACM)	DFUs	Not applicable	Completed	HPTC group achieved significantly better healing outcomes, with 85.71% of patients exhibiting ≥50% wound size reduction at 4 weeks compared with 50% of patients in the dHACM group.	NCT06470087
Noxsano bandage	Hydrogel‐based dressing designed to deliver controlled doses of nitric oxide (NO) to chronic wounds	Diabetic wound	I/II	Completed	Reduction in wound size and evaluation of granulation tissue formation	NCT04123093
UC‐MSCs gel		Difficult to healing of skin ulcers	I	Completed	Not reported	NCT02685722
Autologous PRP gel and PRP injection	PRP	Chronic ulcer	I	Completed	Not reported	NCT03026855
Galnobax	Gel formulation of esmolol hydrochloride	DFUs	I/II	Completed	A safe novel treatment for DFU.	NCT01113515
Allogeneic adipose‐derived MSCs	Fibrin solution containing adipose‐derived MSCs	DFUs	I/II	Completed	Not reported	NCT02361931
Human umbilical cord secretome	Human umbilical cord secretome	Trophic ulcers	I	Completed	Not reported	NCT05777213
Stem cell conditioned media	Conditioned media	Chronic ulcer	I	Completed	Not reported	NCT04134676
Bone marrow mononuclear cells vs MSCs	Cells	Diabetic patients with chronic limb ischemia	I/II	Completed		NCT05631444
Cellular and tissue‐based therapy registry (CTPR)	Cell‐based products			Unknown status		NCT02322554
Stromal Vascular Fraction From Lipoaspirate	Stromal vascular fraction	Chronic nonhealing wound	I	Unknown status	Not reported	NCT03882983
NeoThelium FT	Dehydrated wound covering derived from donated human placental tissue, a dual‐layer membrane with amnion and chorion combination layers	Pressure injuries	Not applicable	Not yet recruiting	Not reported	NCT06918548
NeoThelium FT	Dehydrated wound covering derived from donated human placental tissue, a dual‐layer membrane with amnion and chorion combination layers.	Chronic DFUs	Not applicable	Not yet recruiting	Not reported	NCT06938685
Lipofilling	Autologous transplantation of fat tissue	Chronic lower leg ulcers	Not applicable	Completed	Not reported	NCT05509673
DermGEN	Human acellular dermal matrix (Allograft)	Chronic diabetic wounds	Not applicable	Not yet recruiting	Previous results: 82% of participants with DFU treated with a single application of DermGEN achieved complete wound closure between 2 and 8 weeks	NCT06227520
MicroMatrix and Cytal wound matrix 2‐layer plus NPWT	MicroMatrix and Cytal wound matrix 2‐layer plus NPWT	PUs stage III, pressure ulcer, stage IV, pressure ulcer	Not applicable	Completed	Not reported	NCT03283787
ABCB5‐positive stem cells	MSCs	Chronic venous ulcer	I/II	Completed	Wound size reduction of 63% at 12 weeks and early relief of pain	NCT03257098
Allogeneic ABCB5‐positive dermal mesenchymal stromal cells	Mesenchymal stromal cells	Chronic venous ulcer	IIb	Active, not recruiting	Not available	NCT04971161
Allogeneic ABCB5‐positive dermal mesenchymal stromal cells	Mesenchymal stromal cells	Chronic venous ulcers	III	Recruiting	Not available	NCT06489028
Autologous bone marrow stem cells	Infusion of autologous bone marrow stem cells after wound debridement.	Type IV PUs Chronic wounds Spinal cord injury	I/II	Completed	In 19 patients (86.36%), the PUs treated with BM‐MNCs had fully healed after a mean time of 21 days.	NCT01572376
Dermal regeneration photosynthetic matrix (DRPM)‐HULK	Photosynthetic scaffolds containing microalgae	Full‐thickness skin wounds	Early phase I	Completed	Implanted scaffolds did not trigger any deleterious local or systemic immune responses in a 90 days follow‐up, allowing full tissue regeneration in humans.	NCT03960164
Bone marrow stem cells + MSCs	Bone marrow stem cells + MSCs (CD90+)	DFUs	II	Completed	18 patients showed wound healing after 45 weeks.	NCT01065337
Curexcell	Primed/activated monocytes, neutrophils and lymphocytes derived from whole blood unit	Chronic wounds		Completed	The use of a macrophage suspension is a safe and effective therapeutic strategy that shortens the healing period, reduces risk of complications and morbidity.	NCT02742844
Miro3D wound matrix	Acellular porcine‐derived wound matrix	DFUs VLUs PUs	Not applicable	Recruiting	Not available	NCT06939673
PDGF‐B/Ad5	Single injection of PDGF DNA in an adenoviral vector.	Chronic VLUs	I	Completed	Not available	NCT00000431
Marigen wound dressing	Fish skin ECM	DFUs, VLUs, PUs	Not applicable	Completed	Not available	NCT01348581
DermGEN	DermGEN is a decellularized donated human tissue.	Diabetes type I, diabetes type II	I	Completed	Not available	NCT02184455
Meso wound matrix	Acellular scaffold	DFUs	Not applicable	Completed	Not available	NCT04182451
CellMist	Autologous stem cell spray	Deep second degree burns	I	Unknown	Not available	NCT04890574
AUP1602‐C	Genetically engineered L. lactis	DFUs	I/II	Completed	Not available	NCT04281992
NanoSALV	Antimicrobial wound dressing	Chronic wounds	Not applicable	Completed	Not available	NCT05619237

*Data sources*—clinical registration website (ClinicalTrials.gov).

The role of EVs in modulating immune responses is increasingly recognized. Their cargo composition and biological impact are highly dynamic, shaped by the microenvironment and cellular context [337]. Emerging evidence suggests that EVs serve as both sensors and regulators of inflammation—capable of augmenting or suppressing immune responses depending on the physiological or pathological context. While most insights into their immunomodulatory roles have been derived from in vitro studies or exogenously applied EVs, the translation of these findings into physiologically relevant in vivo models remains essential. The lack of standardized dosing regimens and the technical limitations of current EV isolation methods further complicate the interpretation of their functional relevance [338]. Still, their consistent influence on both innate and adaptive immunity across multiple studies underscores their potential as key modulators of immune function. EVs may act as first responders to tissue injury—initiating, shaping, and resolving immune responses through their finely tuned cargo [339]. As isolation techniques advance toward single‐vesicle resolution, a more precise understanding of how specific EV populations influence distinct immune cell types will likely emerge. Despite current challenges in standardization, scalability, and regulatory approval, engineered EVs offer an exciting therapeutic platform. Their ability to be modified for targeted delivery and their inherent biocompatibility position them as promising agents in immune‐based therapies. Moving forward, the development of genetic models for in vivo EV tracking, and the use of GMP‐compliant production protocols, will be critical for realizing their clinical potential.

MSC‐derived EVs accelerate wound healing by modulating inflammation, promoting angiogenesis, enhancing epithelial and fibroblast proliferation and migration, and supporting ECM remodeling. However, their clinical translation faces challenges due to variability in therapeutic outcomes influenced by the MSC source, EV concentration, dosing regimen, administration route, and the recipient's inflammatory state. Addressing these issues requires standardized GMP‐compliant isolation protocols and thorough molecular characterization, including particle size, surface markers, and bioactive cargo. The heterogeneity of MSC‐EVs—including exosomes, microvesicles, and apoptotic bodies—demands precise classification and functional analysis. Optimizing dosing, clearance kinetics, and in vivo tracking is essential for clinical use. Identifying markers to distinguish functional from nonfunctional EVs will further refine therapeutic applications. While significant progress has been made, MSC‐EVs remain in early clinical stages. Advancing their use will depend on robust single‐molecule characterization, scalable production, and continued mechanistic and clinical research. With these efforts, MSC‐EVs hold strong potential as a next‐generation, cell‐free therapy for skin regeneration and chronic wound treatment.

## 6 Translational and Clinical Considerations of Immunomodulatory Therapies

In recent years, significant attention has been directed toward translating immunomodulatory strategies from bench to bedside in the context of chronic wound healing. Encouraging preclinical data have laid the groundwork for translational efforts, with many of these therapies now progressing into early‐ and mid‐phase clinical trials. This has resulted in a growing number of clinical trials exploring innovative therapies aimed at modulating the immune response to restore effective healing. These interventions encompass a diverse range of therapeutic modalities, including stem cell‐based treatments, bioengineered scaffolds, cytokine delivery systems, and EV‐based formulations—each designed to target specific aspects of immune dysfunction observed in chronic wounds. Among these, MSCs and their derivatives (e.g., MSC‐conditioned media or MSC‐derived EVs) are being evaluated for their ability to reduce inflammation, promote macrophage polarization toward the reparative M2 phenotype, and stimulate tissue regeneration. Trials involving topical or injectable MSC products have reached phase I to phase III stages for conditions such as DFUs and burn wound, reflecting both safety and growing evidence of clinical benefit. Similarly, EV‐based therapies, though in earlier clinical stages, have gained traction due to their capacity to deliver immunomodulatory cargo such as miRNAs, cytokines, and enzymes while avoiding some of the complexities of cell‐based therapies. Table 5 summarizes currently ongoing clinical trials exploring these biological and nonbiological approaches, reflecting the growing interest and investment in translating these therapies to clinical practice.

MSCs are among the most extensively studied cell‐based therapies for chronic wounds. They exert potent immunomodulatory effects through the secretion of anti‐inflammatory cytokines, growth factors, and EVs. In clinical trials, MSCs derived from bone marrow, adipose tissue, or umbilical cord have demonstrated promising results in enhancing re‐epithelialization, angiogenesis, and reducing inflammation, particularly in DFUs and VLUs. However, variability in cell source, dosage, viability, and delivery methods remain significant translational hurdles. Additionally, issues related to scalability and regulatory classification continue to challenge the widespread clinical adoption of MSC‐based therapies. EVs derived from MSCs or immune cells represent a cell‐free alternative that carries bioactive molecules such as miRNAs, cytokines, and proteins capable of immunomodulation. Their nano‐size allows for better tissue penetration, reduced immunogenicity, and lower risk of tumorigenicity compared with whole‐cell therapies. Clinical interest in EVs is rising due to their potential to mimic the regenerative effects of parent cells while offering improved safety and stability profiles. Early‐phase clinical trials are exploring their use in chronic ulcers and postsurgical wound healing, though standardization of isolation, characterization, and dosing remains a key challenge.

Biomaterial‐based therapies such as hydrogels are being developed not only as structural scaffolds but also as bioactive platforms that modulate immune cell behavior. Immunomodulatory biomaterials can direct macrophage polarization, enhance fibroblast function, and support angiogenesis. Clinical trials are evaluating both natural (e.g., collagen, alginate) and synthetic (e.g., PEG‐based) hydrogels, either alone or in combination with other therapies. The biocompatibility, degradation kinetics, and immunological impact of these materials are critical for their success in chronic wound treatment. Targeted cytokine delivery aims to correct immune dysregulation in chronic wounds. For example, IL‐10 and TGF‐β have shown promise in promoting anti‐inflammatory macrophage phenotypes and enhancing matrix remodeling. Topical formulations of these cytokines or gene therapy approaches are under evaluation for their ability to reduce chronic inflammation and stimulate granulation tissue formation. However, short half‐life, off‐target effects, and the need for localized and sustained delivery pose ongoing challenges for clinical translation. Excessive protease activity, particularly from MMPs, is a hallmark of chronic wounds and contributes to ECM degradation and cytokine inactivation. Protease inhibitor therapies aim to rebalance this proteolytic environment, preserving key matrix components and signaling molecules essential for healing. Clinical studies are testing topical protease modulators, including ORC/collagen dressings, which have demonstrated some success in reducing wound size and improving healing rates. A significant translational challenge remains in identifying optimal candidates for these therapies and determining precise dosing to avoid inhibiting beneficial protease activity required during early wound phases.

## 7 Conclusion and Future Outlook

Chronic wounds represent a significant clinical challenge due to their persistent inflammation, impaired tissue regeneration, and high patient morbidity. A growing body of evidence underscores the pivotal role of immune dysregulation in the pathogenesis of chronic wounds, highlighting immunomodulation as a promising therapeutic strategy. Advances in cellular therapies, EVs, cytokine‐targeted treatments, and bioengineered materials have demonstrated encouraging results in modulating the wound microenvironment toward healing. However, translating these therapies into clinical practice remains hindered by biological heterogeneity, patient comorbidities, and regulatory complexities. Despite major advances in our understanding of immune involvement in wound healing, we remain far from achieving truly effective, personalized immunomodulatory therapies for chronic wounds. The complexity of the chronic wound microenvironment—driven by persistent inflammation, immune dysregulation, and patient‐specific variables such as diabetes or vascular insufficiency—makes therapeutic intervention especially challenging. Current clinical strategies largely focus on symptomatic management rather than mechanistically targeted repair, underlining the translational gap. While the mechanistic understanding of immune cell dynamics in wound healing has expanded—especially the roles of macrophage polarization, T‐cells regulation, and stromal‐immune interactions—this has yet to translate into widely adopted clinical solutions. Recent studies and clinical trials, however, signal promising directions. For instance, allogeneic MSCs like Alofisel (NCT05974280) are already approved in Europe for complex perianal fistulas, showcasing the immunomodulatory potential of MSCs in tissue repair contexts. Similarly, EVs derived from MSCs and engineered to carry miRNAs are entering early‐stage trials for DFUs (e.g., NCT05078385), offering cell‐free, scalable alternatives. Bioengineered hydrogels that respond to local cytokine levels and release immunoregulatory agents in a controlled manner represent another translational leap, with products like PuraPly AM (NCT03070925) and Grafix (NCT01596920) showing early efficacy in wound clinics.

Despite the promising advancements in immunomodulatory therapies for chronic wound healing, significant gaps persist between preclinical research and clinical application. One major challenge is the incomplete understanding of the complex immune dysregulation underlying chronic wounds. Unlike acute wounds, chronic wounds exhibit prolonged inflammation and immune dysfunction, often compounded by patient‐specific factors such as diabetes, vascular insufficiency, or aging. However, the precise mechanisms—such as the roles of distinct immune cell subsets, their temporal activation, and crosstalk with nonimmune cells—remain insufficiently characterized. This lack of mechanistic clarity hampers the rational design of targeted therapies. Furthermore, most preclinical studies rely on acute wound models in healthy animals, which fail to replicate the chronic inflammatory environment seen in human patients. Consequently, many therapeutic candidates that show efficacy in animal models do not translate effectively to human trials. Chronic wounds are not a uniform disease entity but encompass diverse types such as DFUs, VLUs, and PUs—each with distinct pathophysiological mechanisms and immune signatures. DFUs are driven by ischemia, neuropathy, and hyperglycemia‐induced immune dysfunction, often characterized by impaired macrophage activation and neutrophil persistence. VLUs result primarily from chronic venous insufficiency and local tissue hypoxia, with sustained neutrophilic inflammation and high protease levels. PUs are caused by prolonged mechanical pressure, leading to localized ischemia‐reperfusion injury and sterile inflammation. This heterogeneity complicates clinical trial design, patient recruitment, endpoint selection, and therapeutic generalization. Immunomodulatory strategies that benefit one wound type may not translate across others due to differing inflammatory dynamics and healing trajectories. Moreover, chronic wound patients often have systemic comorbidities—diabetes, peripheral vascular disease, renal insufficiency, obesity, and aging‐related immunosenescence—that profoundly alter immune responses and tissue repair. Hyperglycemia disrupts neutrophil function and suppresses M2 macrophage polarization. Chronic kidney disease and aging impair DC function and T‐cells activation. Vascular diseases reduce perfusion and immune cell trafficking. These factors influence both the baseline immune profile and the response to immunomodulatory therapy, resulting in variability in clinical outcomes. Moreover, immune‐suppressive states (e.g., in elderly or diabetic patients) may limit the effectiveness of proregenerative interventions, necessitating stratification or combination therapies. Cell‐based and EVs‐based therapies are biologically complex products that present unique challenges for clinical translation: standardization of MSCs or EVs products across donors, tissue sources, and culture conditions is difficult; potency assays and batch release criteria for these biologics are not yet universally established; and storage, transport, and shelf‐life of living cells or EVs require rigorous quality control systems. Regulatory categorization varies globally, for example, cell therapies may be classified as advanced therapy medicinal products in the European Union or biologics under Food and Drug Administration rules in the USA, each with distinct trial and approval pathways. This complexity increases development costs, slows regulatory approval, and hinders scalability, especially for allogeneic or off‐the‐shelf cell and EV products.

Additionally, clinical trials often lack predictive biomarkers or robust immunological endpoints, relying primarily on wound closure as the main outcome measure, which may not fully capture early immunomodulatory effects. Without reliable markers for immune activity or patient stratification tools, therapies may fail in clinical trials despite having mechanistic potential. Moreover, high production costs, scalability issues, and limited engagement from industry stakeholders further slow the transition of these therapies from bench to bedside. To bridge the gap between preclinical findings and clinical application, several research tools and platforms are being developed. These include 3D wound models, organ‐on‐chip systems, and high‐resolution immunoprofiling techniques such as scRNA‐seq and multiplex imaging. These tools enable better characterization of the immune landscape within chronic wounds and help identify therapeutic windows and patient‐specific targets. Moreover, the integration of omics‐based analyses (transcriptomics, proteomics, and metabolomics) in clinical trial design is expected to refine patient stratification and optimize treatment efficacy. Altogether, these clinical and translational research efforts represent a paradigm shift toward precision medicine in wound care. While challenges remain in standardizing outcome measures and ensuring long‐term safety, the momentum behind immunomodulatory therapies continues to grow, paving the way for more effective and personalized interventions for chronic wounds. Altogether, the integration of precision medicine approaches—such as patient‐specific immune profiling, omics technologies, and advanced wound models—is essential to overcome these translational barriers. Bridging this gap requires a multidisciplinary effort to align scientific discovery with clinical feasibility, regulatory compliance, and therapeutic efficacy. The evolving landscape of wound care is poised for transformation with advancements in biomaterials that enable precise immunomodulation. Future strategies may include smart wound dressings capable of targeted pathogen elimination and controlled macrophage polarization. Metal‐organic frameworks (MOFs) are emerging as promising carriers for therapeutic agents, offering high surface area, tunable pore sizes, and controlled drug release capabilities. MOFs loaded with bioactive molecules, such as titanium carbide MXene (Ti_3_C_2_), have demonstrated antibacterial properties and enhanced wound‐healing efficacy. Interdisciplinary collaboration between clinicians, bioengineers, and materials scientists is essential for translating these innovations from the laboratory to clinical practice. As wound‐healing research advances, the integration of bioactive dressings, tissue engineering, and stem cell therapies will accelerate regenerative outcomes. Technologies such as 3D printing, nanotechnology, and artificial intelligence will drive personalized treatment strategies, while telemedicine and remote monitoring will improve healthcare accessibility. The path forward lies in refining our mechanistic understanding while simultaneously building smarter, modular delivery systems. While we are still a significant distance from routine, immune‐targeted therapies for chronic wounds, the field is steadily advancing. With sustained cross‐disciplinary collaboration, patient‐tailored immunotherapies are a realistic goal on the near horizon. Ultimately, a deeper understanding of immune mechanisms in wound repair, coupled with innovation in translational strategies, holds the potential to revolutionize the treatment landscape for chronic wounds.

## Author Contributions

All authors contributed to the conceptualization and writing of the manuscript. M.R. and M.Z.I. conducted the literature review and drafted the initial manuscript. A.S.K. and T.B. provided critical revisions and edits. All authors read and approved the final manuscript.

## Ethics Statement

The authors have nothing to report.

## Conflicts of Interest

The authors declare no conflicts of interest.

## Data Availability

The authors have nothing to report.
